# Enhancement of Anti-Neoplastic Effects of MGN-3/Biobran Against Solid Ehrlich Carcinoma-Bearing Mice via Lipidic Nanoparticle Based Targeted Drug Delivery System

**DOI:** 10.3390/ijms27177953

**Published:** 2026-09-07

**Authors:** Zeinab A. Alerksosy, Mamdooh H. Ghoneum, Mai Alaa El-Dein, Sarah Yahia, Ibrahim M. El-Sherbiny, Nariman K. Badr El-Din

**Affiliations:** 1 Zoology Department, Faculty of Science, Mansoura University, Mansoura 35516, Dakahlia Governorate, Egypt; zeinabelmotwaly@gmail.com (Z.A.A.); nkbadreldin@mans.edu.eg (N.K.B.E.-D.); 2 Department of Surgery, Charles Drew University of Medicine and Science, Los Angeles, CA 90059, USA; 3 Department of Surgery, University of California, Los Angeles, Los Angeles, CA 90095, USA; 4 Nanomedicine Research Labs, Center for Materials Science (CMS), Zewail City of Science and Technology, 6th October City 12578, Giza, Egypt; smoustafa@zewailcity.edu.eg (S.Y.); ielsherbiny@zewailcity.edu.eg (I.M.E.-S.)

**Keywords:** MGN-3, Lipidic nanoparticle, cell cycle, apoptosis, TNF-α, IL-6

## Abstract

MGN-3 (Biobran), a denatured hemicellulose compound derived from rice bran, possesses potent immunomodulatory and antitumor activity; however, its clinical application is constrained by non-specific tissue distribution, rapid systemic elimination, and poor cellular uptake. To overcome these pharmacological limitations, we engineered MGN-3-loaded lipid nanoparticles (MGN-3.LNPs) formulated with bioactive cinnamon and avocado oils to optimize targeted drug delivery against solid carcinoma. Mice bearing subcutaneous Ehrlich Ascites Carcinoma (EAC) solid tumors received free MGN-3, plain lipid nanoparticles (plain LNPs), or MGN-3.LNPs three times weekly from day 8 to day 26 post-inoculation. The administration of MGN-3.LNPs achieved superior tumor volume suppression (95.00%) compared to free MGN-3 (69.00%) and plain LNPs (63.00%) (*p* < 0.0001). Mechanistically, MGN-3.LNPs effectively inhibited cancer cell proliferation by suppressing Ki-67 expression while promoting expression shifts that strongly suggest the engagement of mitochondrial-mediated apoptotic signaling, including upregulation of tumor protein p53, Caspase-3, Caspase-9, poly(ADP-ribose) polymerase (PARP), and cytosolic cytochrome c (Cyt c), alongside an elevated Bax/Bcl-2 ratio and reduced 8-hydroxy-2′-deoxyguanosine (8-OHdG) levels. Flow cytometric analysis confirmed that MGN-3.LNPs induced marked G0/G1 cell cycle arrest and promoted sub-G1 apoptotic cell accumulation, which was corroborated by Annexin V/propidium iodide (Annexin V/PI) staining and semiquantitative histopathological evaluation. Furthermore, MGN-3.LNPs downregulated the gene expression of proinflammatory cytokines tumor necrosis factor-alpha (TNF-α) and interleukin-6 (IL-6) while restoring redox homeostasis in tumor tissues. Overall, lipidic nanoencapsulation significantly enhances the therapeutic efficacy of MGN-3 against solid tumors through superior nanoscale tissue penetration, prolonged retention, and synergistic lipid–drug bioactivity.

## 1. Introduction

Female breast cancer remains the most commonly diagnosed malignancy and a leading cause of cancer-related mortality globally [1]. Despite significant advancements in surgical, radiotherapeutic, and systemic modalities, conventional chemotherapies are frequently constrained by severe systemic toxicity, drug resistance, and suboptimal tumor selectivity [2]. While natural bioactive products offer promising multi-targeted therapeutic potential [3], their clinical utility is severely limited by poor bioavailability, low aqueous solubility, and inadequate tissue accumulation at tumor sites. Targeted nanocarrier platforms address these biopharmaceutical hurdles by controlling drug release kinetics, facilitating site specific delivery, and enhancing therapeutic efficacy while reducing off-target adverse effects [4]. To evaluate these nanotherapeutic strategies in vivo, we utilized Ehrlich Ascites Carcinoma (EAC), a well-established transplantable murine mammary adenocarcinoma model derived from a spontaneous mouse breast tumor, as our preclinical platform due to its distinct mammary lineage, aggressive growth kinetics, and shared biological traits with solid breast carcinoma [5,6].

Multifunctional lipid-based nanoparticles (LNPs) are widely investigated for cancer therapeutics due to their high tumor cell internalization, capacity to inhibit disease progression, and low off-target toxicity [7]. Unlike conventional polymeric, metallic, inorganic, or liposomal nanocarriers, which are frequently constrained by inflammatory acidic degradation byproducts, prolonged tissue accumulation, concentration-dependent organ toxicity, and physical instability, LNPs offer superior biocompatibility, high physical stability, and benign metabolic degradation. In this formulation, a specialized LNP matrix composed of cinnamon and avocado oils was developed to encapsulate MGN-3. Beyond functioning as a solubilizing delivery vehicle, this lipid matrix rich in cinnamaldehyde, oleic acid, and α-tocopherol exerts intrinsic antioxidant, anti-inflammatory, and membrane permeabilizing effects. These bioactive lipids may act synergistically with MGN-3 to enhance cellular uptake and potentiate anti-tumor efficacy while minimizing carrier-induced systemic toxicity.

Essential oil (EO)-based nano-emulsions have attracted attention in medicine [8,9,10,11] due to their effective anticancer actions [12]. Decreasing the diameter of droplets to nanometer sizes increases their bioavailability and solubility, thereby leading to the higher permeation of active compounds into blood vessels [13]. Cinnamon essential oil extracted from species of the genus *Cinnamomum* contains cinnamaldehyde as its primary bioactive constituent [14,15], and has demonstrated significant anticancer activity [16]. Furthermore, cinnamon oil nanoemulsions have shown potent efficacy against both colistin-resistant *Klebsiella pneumoniae* and various cancer cell lines [17]. Avocado oil is another EO extracted as a versatile, nutrient-dense oil from the pulp of the avocado fruit, and can be formulated into a nanoemulsion, which involves dispersing it into extremely small droplets (nano-sized) within an aqueous phase [18]. Using the nanoformulation of natural ingredients could represent a method for improving their action as anticancer medicines by improving their stability and bioavailability [17].

MGN-3/Biobran is a natural dietary product manufactured by the enzymatic cleavage of rice bran hemicellulose using carbohydrate-hydrolyzing enzymes derived from shiitake mushrooms [19]. Functioning as a potent biological response modifier (BRM), MGN-3/Biobran stimulates key cellular components of the immune system, particularly dendritic cells (DCs) [20] and natural killer (NK) cells [21,22,23]. Its broad-spectrum anticancer efficacy has been validated across diverse animal cancer models. Mechanistically, MGN-3/Biobran enhances endogenous antioxidant defenses, regulates lipid peroxidation, and mitigates oxidative stress [24]. Furthermore, it protects against chemically induced glandular stomach [25] and hepatic carcinogenesis [26] by suppressing inflammation and triggering apoptosis in malignant cells. Notably, MGN-3/Biobran synergistically augments the therapeutic efficacy of paclitaxel in breast adenocarcinoma [27] and liver metastasis models [28], while also enhancing tumor radiosensitivity in vivo [29]. Furthermore, clinical studies have confirmed its capacity to boost NK cell activity in geriatric subjects and improve patient quality of life [30,31]. We previously demonstrated the antitumor efficacy of unformulated (free) MGN-3/Biobran in an Ehrlich Ascites Carcinoma (EAC) murine model of breast cancer [22]. Building upon those findings, the present study aimed to enhance the therapeutic efficacy of MGN-3/Biobran against tumor growth and cell proliferation through lipid-based nanoencapsulation utilizing a blend of cinnamon and avocado oils. This nanoformulation strategy was designed to improve drug bioavailability, enhance tumor targeting selectivity, and enable sustained drug release. Furthermore, lipid nanoparticles constructed from biocompatible lipids improve the stability of encapsulated bioactive agents while minimizing systemic toxicity [32].

## 2. Results

### 2.1. Characterization of Plain and MGN-3-Loaded Lipidic Nanoparticles (MGN-3.LNPs)

#### 2.1.1. Zeta Size and Potential of Plain Nanoparticles and MGN-3.LNPs

**Figure 1:**
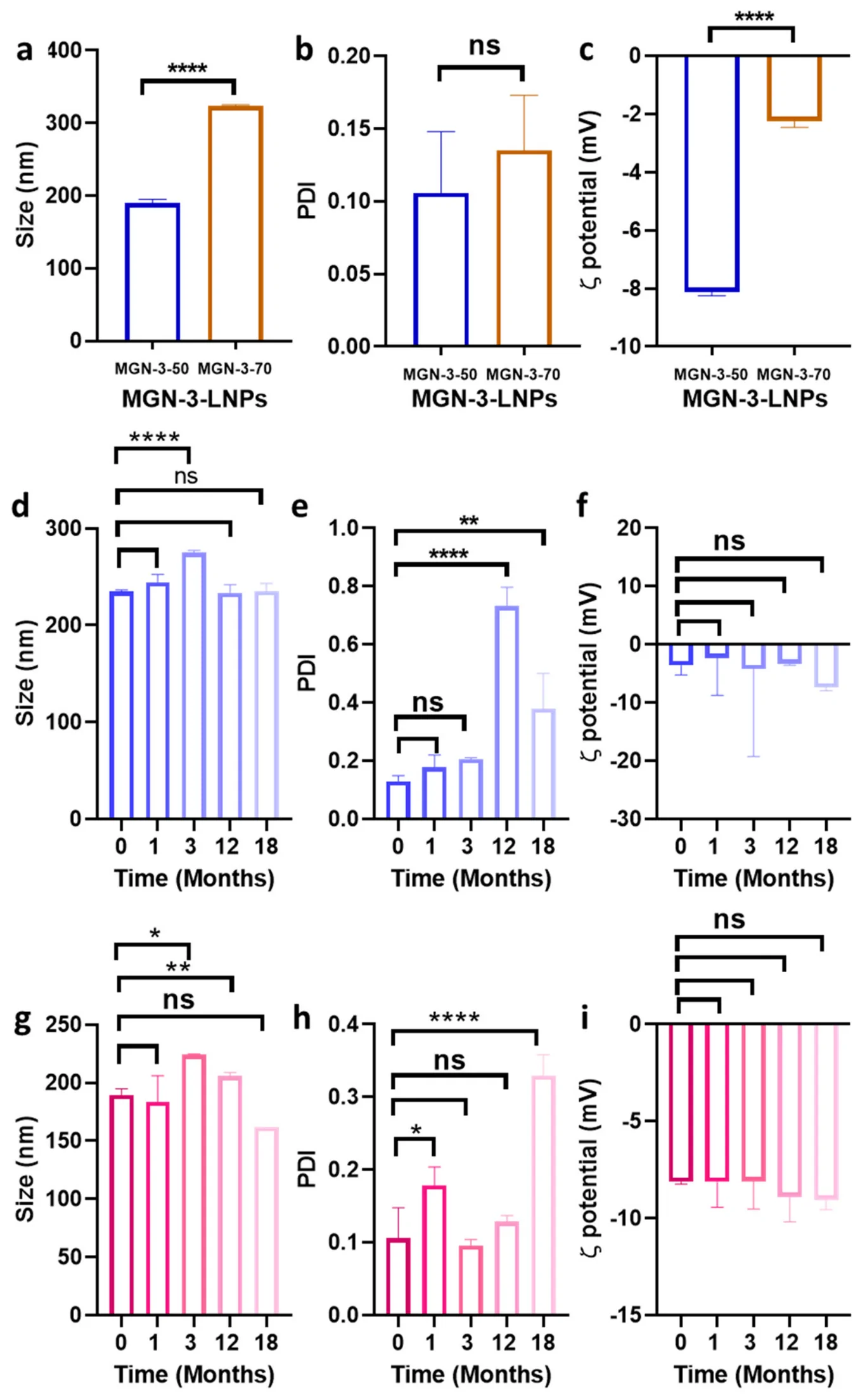
Physicochemical characterization and 18-month storage stability of plain and MGN-3.LNPs. (**a**–**c**) Comparison of hydrodynamic particle size (nm) (**a**), polydispersity index (PDI) (**b**), and zeta (ζ) potential (mV) (**c**) between formulations loaded with 50 mg (MGN-3-50) and 70 mg (MGN-3-70) of MGN-3. (**d**–**f**) Long-term physical stability of plain LNPs stored at 4 °C over an 18-month duration evaluating changes in particle size (**d**), PDI (**e**), and ζ potential (**f**) relative to baseline (Month 0). (**g**–**i**) Long-term physical stability of MGN-3.LNPs (MGN-3-50) stored at 4 °C over an 18–month duration evaluating changes in particle size (**g**), PDI (**h**), and ζ potential (**i**) relative to baseline (Month 0). Values are presented as Mean ± SD. (*, **, ****) indicate statistical significance difference at *p* < 0.05, *p* < 0.01, and *p* < 0.0001 levels, respectively (ns = not significant).

The average size and zeta (ζ) potential of both plain and MGN-3-loaded NPs (MGN-3.LNPs) were examined by Zetasizer. Table 1 shows the plain NP’s size (234.8 ± 9.38 nm) and corresponding surface charge (ζ) (−3.56 ± 1.69 mV). The encapsulation of the drug (MGN-3) at different quantities into lipidic nanoparticles influences the physicochemical characterization. As shown in Table 1 and Figure 1, using 50 mg of the loaded drug improved these characterizations over 70 mg. Where their size decreased, surface charge showed greater negativity. The size of MGN-3.LNPs decreased significantly to 189.7 ± 5.273 nm, with an increase in ζ to −8.22 ± 0.127 mV.

Moreover, Figure 1 shows a small range of PDI for all the prepared samples, indicating good colloidal stability and the good dispersion of the NPs, either freshly prepared or those stored at 4 °C. In addition, the stability of all samples was studied over 1.5 years to test their shelf stability in liquid form. As can be noted from all figures, all parameters remained almost stable, with little changes throughout this long period. These data are indicative of the good stability of the prepared samples.

#### 2.1.2. Transmission Electron Microscope (TEM) Measurements of Plain Nanoparticles & MGN-3.LNPs

**Figure 2:**
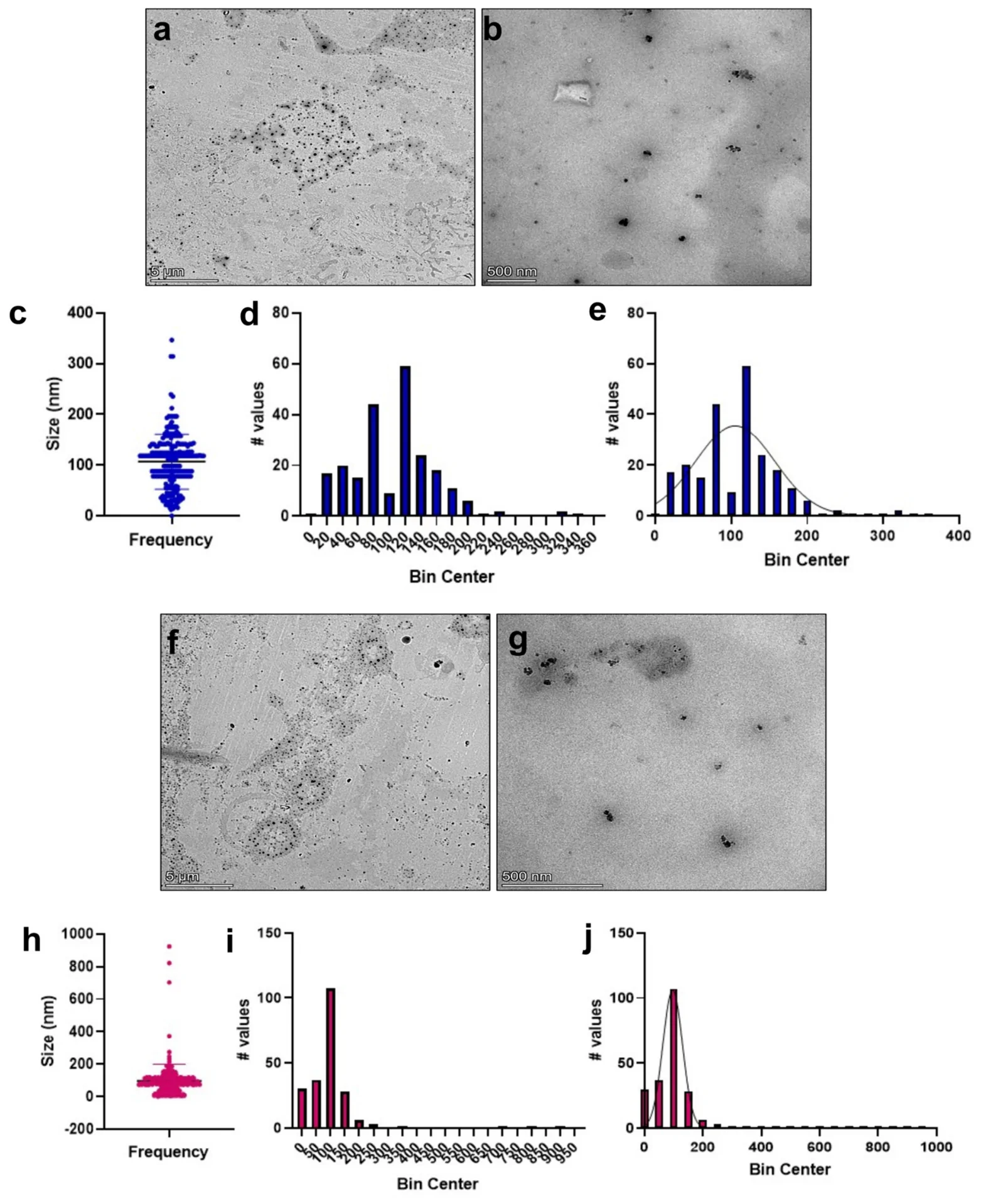
Morphological characterization and particle size distribution of plain and MGN-3-loaded lipid nanoparticles. Transmission electron micrographs of plain LNPs (**a**,**b**) and MGN-3.LNPs (**f**,**g**) at low magnification (scale bar = 5 µm; (**a**,**f**)) and high magnification (scale bar = 500 nm; (**b**,**g**)). Size distribution analysis for plain LNPs (**c**–**e**) and MGN-3.LNPs (**h**–**j**) displaying scatter dot plots (**c**,**h**), frequency histograms (**d**,**i**), and non-linear Gaussian regression curve fits (**e**,**j**). Plain LNPs exhibited a mean size of 106.1±52.2 nm ($R^{2}=0.64$), whereas MGN-3.LNPs demonstrated a mean size of 96.6±33.7 nm ($R^{2}=0.93$). # is the range values. These data indicate that drug encapsulation reduced both mean particle diameter and size polydispersity.

TEM micrographs of NPs are shown in Figure 2a–j. As can be noted, both plain and MGN-3-loaded NPs are spherical in shape. In addition, the micrographs confirm the mean particle sizes of plain and MGN-3.LNPs measured with the TEM particle analysis software (NanoScope Analysis, Veeco Co., Santa Barbara, CA, USA). Morphological size distribution analysis via transmission electron microscopy (TEM) image processing (ImageJ, version 1.53t, National Institutes of Health, Bethesda, MD, USA) revealed that plain lipid nanoparticles (LNPs) exhibited a mean diameter of 106.1±52.2 nm with a broad size distribution ($R^{2}=0.64$). Upon incorporation of MGN-3, the mean particle diameter decreased to 96.6±33.7 nm and displayed a significantly narrower distribution profile ($R^{2}=0.93$). This reduction in both particle size and polydispersity following drug loading aligns with the dimensional trends observed in parallel dynamic light scattering (DLS) analyses (see the Supplementary Materials).

#### 2.1.3. Drug Entrapment Efficiency, Loading Capacity and In Vitro Cumulative Drug Release Study

**Figure 3:**
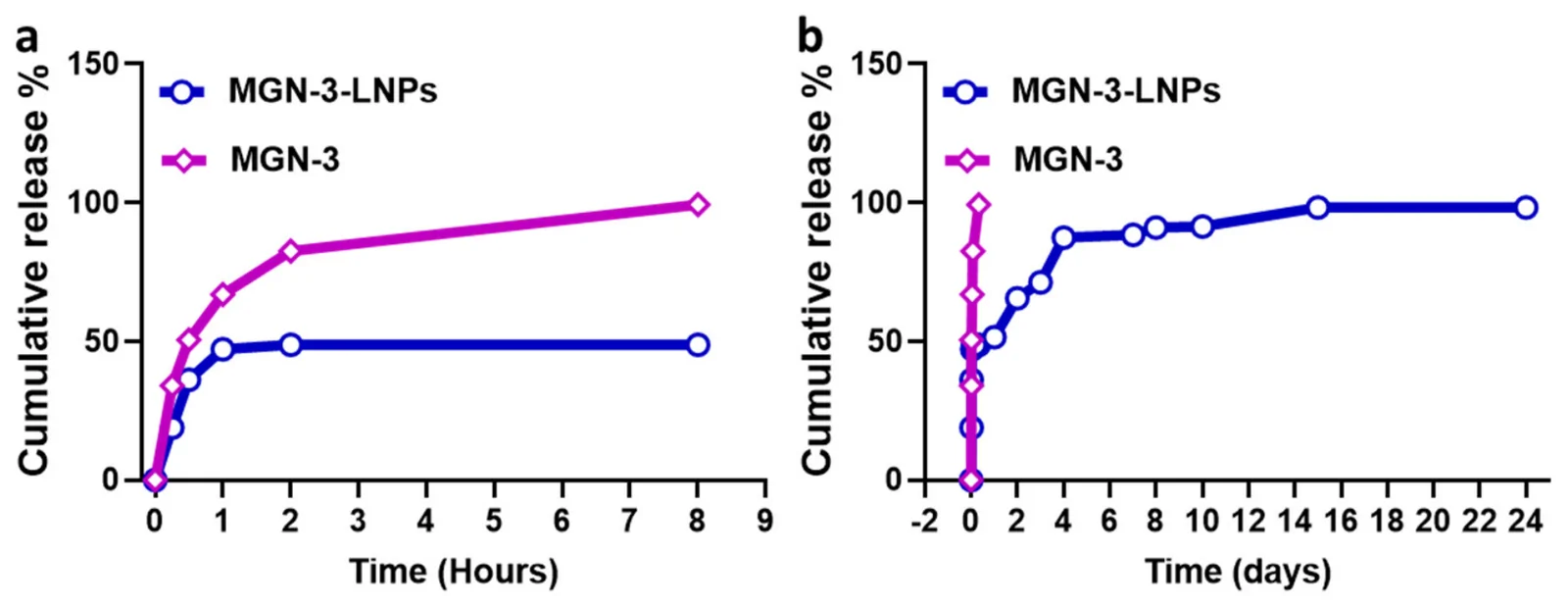
In vitro cumulative drug release of free MGN-3 and MGN-3.LNPs. (**a**) During the first eight hours and (**b**) over 24 days. The study was carried out at 37 °C in PBS with pH 7. Values are average over three triplicates.

The entrapment efficiency (EE%) and loading capacity (LC%) of MGN-3 within the lipid nanoparticles were determined to be 95.99±0.12% and 8.22±0.23%, respectively. The in vitro cumulative drug release kinetics of free MGN-3 and MGN-3-loaded lipid nanoparticles (MGN-3.LNPs) are depicted in Figure 3. Free MGN-3 exhibited rapid burst dissolution, reaching approximately 99% cumulative release within 8 h. Conversely, MGN-3.LNPs displayed a biphasic, sustained release profile characterized by an initial burst release of approximately 45% in the initial hours, followed by continuous, prolonged drug liberation reaching 98% over 24 days (Figure 3). The initial release phase is primarily attributed to surface-adsorbed drug molecules alongside a minor unencapsulated fraction (~4%). Preserving this surface-bound and un-entrapped drug fraction in the final formulation was an intentional design strategy: the immediate burst provision accelerates early therapeutic onset, while the core-encapsulated drug reservoir provides sustained, long-term delivery to maintain antitumor efficacy.

### 2.2. Antitumor Activity of MGN-3-Loaded LNPs Against Solid EAC-Bearing Mice

#### 2.2.1. Changes in Tumor Weight (TW)

**Figure 4:**
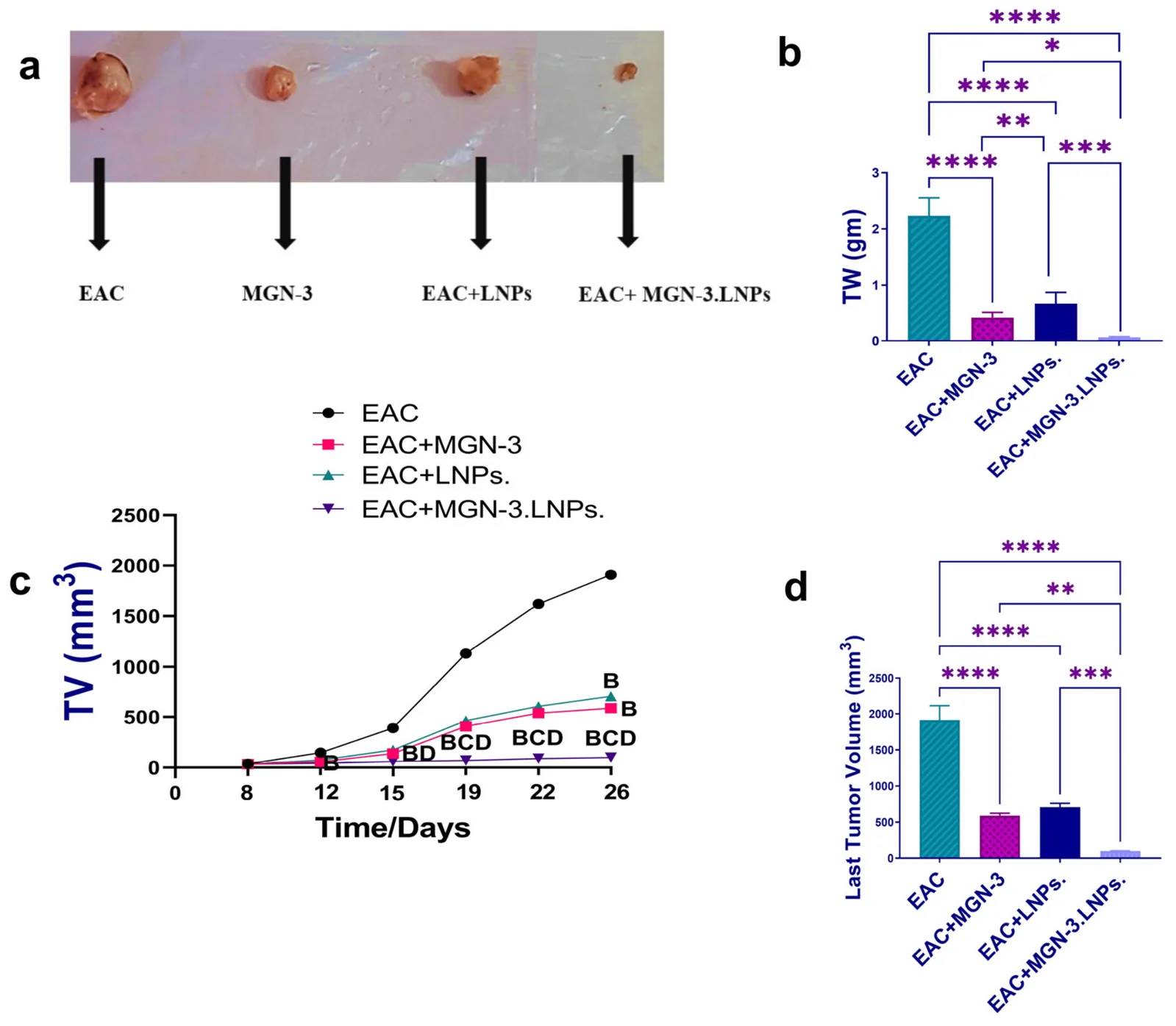
In vivo antitumor efficacy of free MGN-3, plain LNPs, and MGN-3.LNPs in EAC solid tumor-bearing mice. (**a**) Representative photographs of excised solid tumors harvested from each experimental cohort on day 26 post-implantation. (**b**) Final tumor weight (TW, g) measured on day 26. (**c**) Longitudinal tumor volume progression (TV, ${\mathrm{mm}}^{3}$) monitored across the 26-day treatment period. (**d**) Final tumor volume (TV, ${\mathrm{mm}}^{3}$) recorded on day 26. Statistical Analysis and Legend: Data are expressed as Mean ± SEM for each experimental cohort—untreated EAC control (n=9), EAC + free MGN-3 (n=8), EAC+plain LNPs (n=10), and EAC+MGN-3.LNPs (n=14). Statistical comparisons were performed using One-Way ANOVA followed by post-hoc analysis (* p<0.05, ** p<0.01, *** p<0.001, and **** p<0.0001). pB <0.01 vs. untreated EAC group at the corresponding time point. pC <0.05 or p<0.01 vs. EAC + free MGN-3 group at the corresponding time point. pD <0.01 vs. EAC + plain LNPs group at the corresponding time point.

On day 26 post-implantation, solid tumors were harvested, photographed, and weighed to evaluate final tumor weight (TW, g). As illustrated in Figure 4a,b, free MGN-3 administration inhibited tumor growth by 81.19% relative to the untreated control group. Interestingly, treatment with plain LNPs also demonstrated intrinsic tumor suppression, reducing TW by 70.00% compared to untreated controls. Notably, the administration of MGN-3.LNPs exerted superior antitumor activity, achieving a remarkable 97.01% reduction in tumor weight relative to untreated controls, demonstrating the enhanced therapeutic efficacy achieved through nanocarrier encapsulation.

#### 2.2.2. Changes in Tumor Volume (TV)

The untreated Ehrlich Ascites Carcinoma (EAC) control group exhibited progressive tumor volume (TV) expansion throughout the study, reaching $1910\pm 204{\;\mathrm{mm}}^{3}$ by day 26. In contrast, mice treated with free MGN-3 demonstrated significant tumor growth inhibition across time points, resulting in a 69% reduction in TV relative to the control group (p<0.0001). The administration of plain lipid nanoparticles (plain LNPs) also induced a steady deceleration in tumor progression, achieving a 63% decrease in TV compared to untreated controls by day 26 (p<0.0001). Notably, treatment with MGN-3-loaded lipid nanoparticles (MGN-3.LNPs) exerted a profound retardation in tumor growth that became statistically evident as early as day 15 post-inoculation. By day 26, TV inhibition in the MGN-3.LNP cohort reached 95% relative to the untreated EAC control (p<0.0001; Figure 4c,d).

### 2.3. Histopathological Investigation

**Figure 5:**
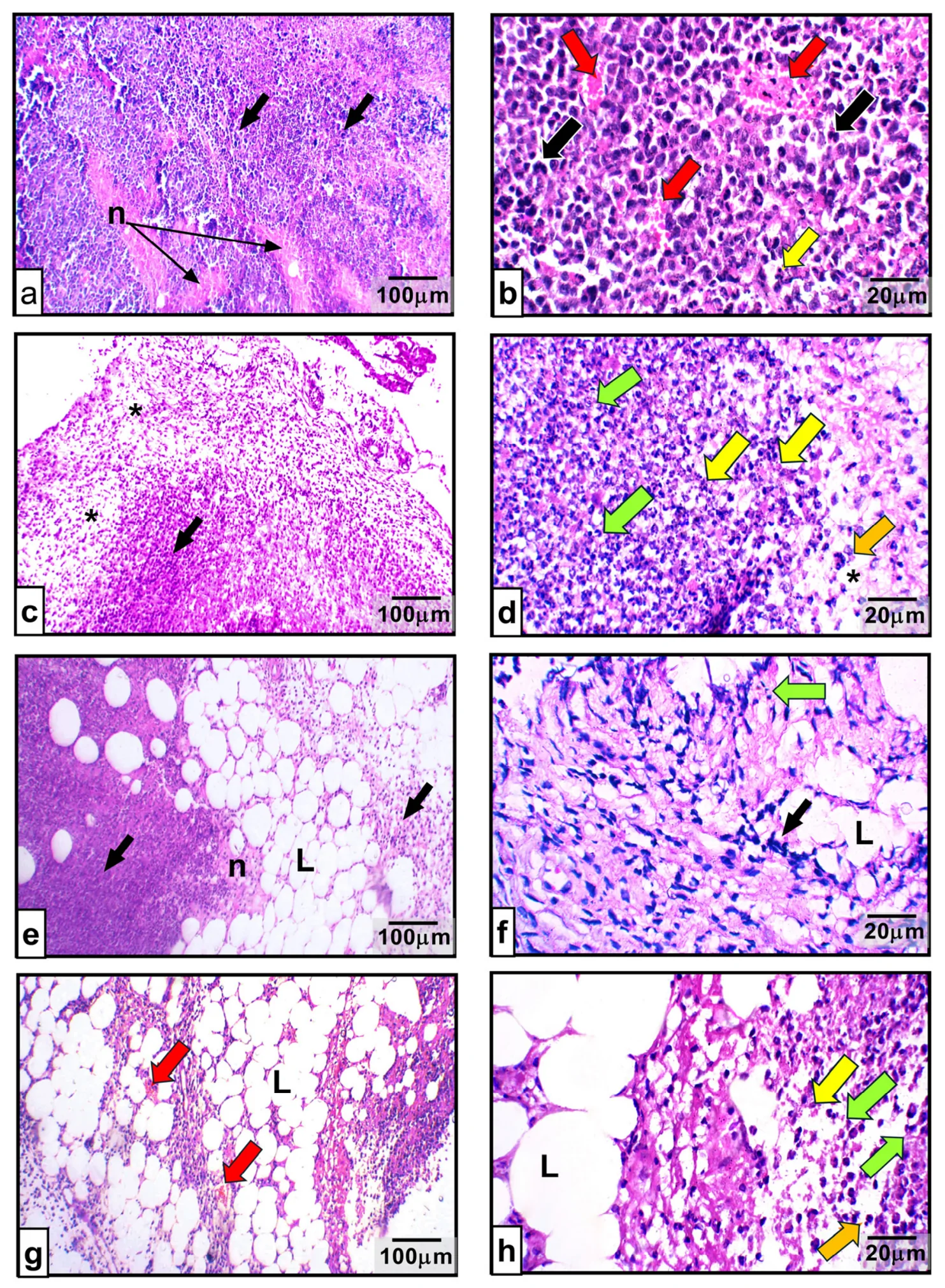
Photomicrographs of sections from EAC-bearing mice of different experimental groups. (**a**,**b**) Untreated EAC group showing solid sheet of active polymorphic Ehrlich neoplasm (black arrow) intruding into coagulative necrosis areas (n), few leucocytes (yellow arrow), and blood cells (red arrow). (**c**,**d**) The EAC+MGN-3 group displaying a large degenerative area (*) in between the neoplasm (black arrow), apoptotic neoplastic cells with pyknotic nuclei (green arrow) or karyorrhexes nuclei (orange arrow), and leucocytic infiltration (yellow arrow). (**e**,**f**) EAC+LNPs group exhibiting lipidic degeneration (L) between the neoplasm (black arrow), as well as a few necrotic foci (n) and a few apoptotic cells (green arrow). (**g**,**h**) EAC+MGN-3.LNPs group illustrating distinct lipidic degenerative regions (L) interspersed throughout tumor tissue, scattered leukocytes (yellow arrow), blood cells (red arrow); and many apoptotic neoplastic cells exhibiting pronounced pyknotic (green arrow) or karyorrhectic nuclei (orange arrow). (H&E stain (hematoxylin and eosin), 100× (**left panel**) and 400× (**right panel**)).

For the untreated EAC group, tumor tissues were found to infiltrate the muscle layer and form solid sheets of undifferentiated tumor cells with a limited number of leucocytes, along with aggregated patches of high-grade viable anaplastic malignancy. In addition, coagulative ischemic necrosis is detectable between the viable pleomorphic neoplastic sheets (Figure 5a,b). The EAC+MGN-3 group exhibited a substantial reduction in the density of neoplastic cells, which was pervaded by a significant number of apoptotic cells with deep eosinophilic cytoplasm, pyknotic nuclei, and a substantial number of leucocytes (Figure 5c,d). Plain lipidic nanoparticle administration to the EAC+LNPs group led to a decline in the density of the tumor neoplasm that was accompanied by the presence of numerous lipid vacuoles in between them (Figure 5e,f). Interestingly, the EAC+MGN-3.LNPs group depicted a substantial degeneration in neoplastic cell proliferation, as well as marked leucocytes infiltration, abundant apoptotic neoplastic cell residues with apparent karyorrhexis or pyknotic nuclei, and marked fatty degeneration (Figure 5g,h).

The semiquantitative histopathological scores taken in between the treatment sets is summarized in this table as follows: (−) is nil, (+) is low levels observed, (++) is moderately observed, and (+++) is highly observed (Table 2).

### 2.4. Immunohistochemical Detection of Ki-67 Expression in Tumor Tissues

**Figure 6:**
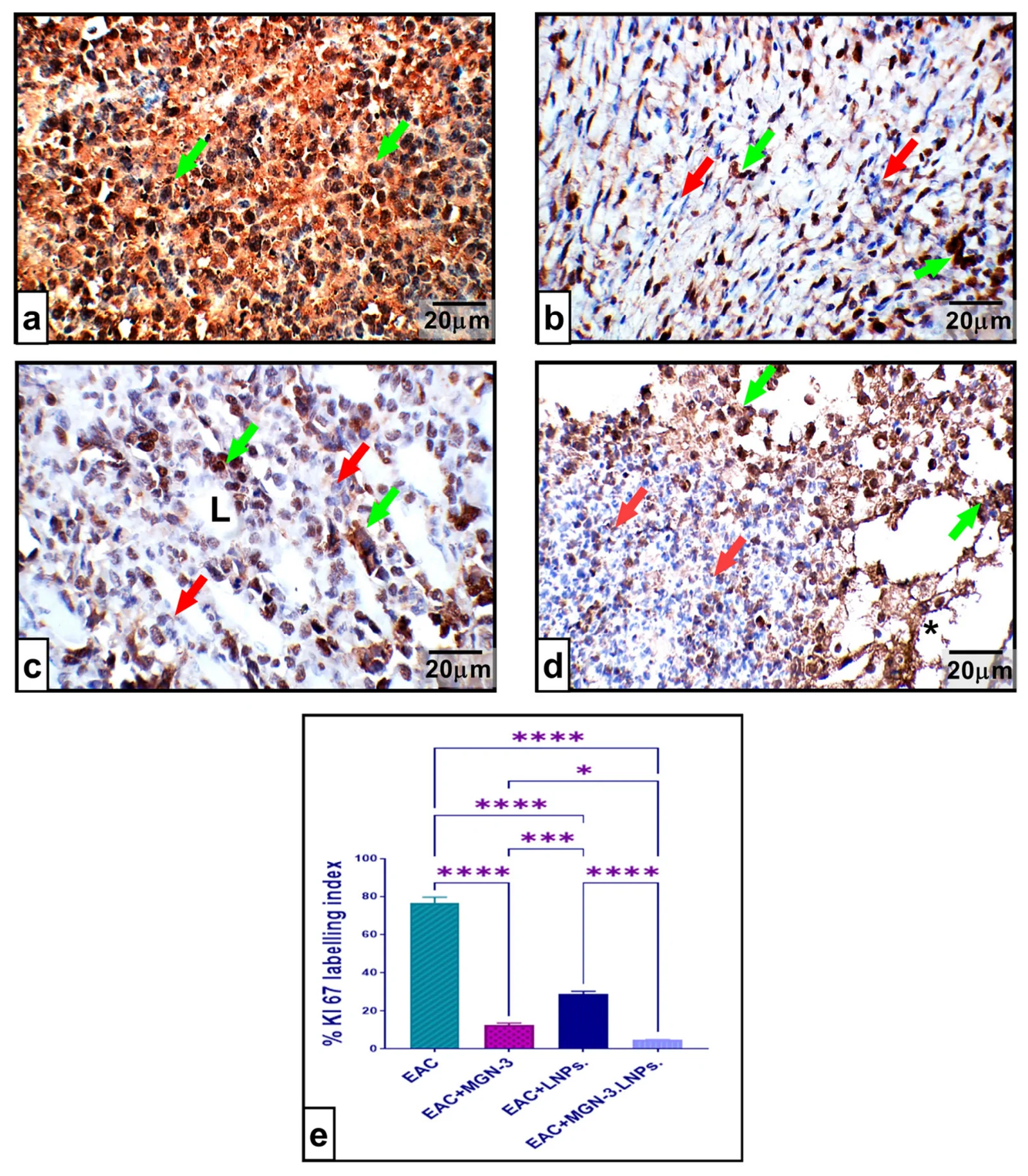
Photomicrographs of tumor sections from the different groups immunostained for Ki-67 protein expression. (**a**) Untreated EAC group showing the overexpression of the Ki-67 protein in the labeled nuclei of the neoplastic cells (green arrow). (**b**) EAC+MGN-3 group showing a decrease in Ki-67 protein expression in the labeled nuclei of the neoplastic cells (red arrow). (**c**) EAC+LNPs group showing moderate nuclear Ki-67 expression in neoplastic cells (green arrow) adjacent to regions of lipidic degeneration (L). (**d**) EAC+MGN-3.LNPs group illustrating weak Ki-67 protein expression in the labeled nuclei (green arrows) between the neoplastic cells (red arrows) and a large degenerative area (*). (**e**) Ki-67 labeling index. (*, ***, ****) indicate statistical significance between groups at *p* < 0.05, *p* < 0.001 and *p* < 0.0001 levels, respectively. (immunoperoxidase, 400×; scale bar, 20 μm).

As illustrated in Figure 6a–e, tumor tissues from untreated EAC-bearing control mice exhibited robust nuclear expression of the Ki-67 protein (Figure 6a). Treatment with plain LNPs significantly reduced the percentage of Ki-67-positive nuclei by 62.13% (*p* < 0.01; Figure 6b), whereas free MGN-3 achieved an 83.36% reduction (*p* < 0.01; Figure 6c). Notably, the administration of MGN-3.LNPs induced the most pronounced suppression, decreasing Ki-67 nuclear expression by 93.73% (*p* < 0.01; Figure 6d) and leaving only weak, sporadic immunoreactivity across the remaining cells.

### 2.5. Cell Cycle Analysis

**Figure 7:**
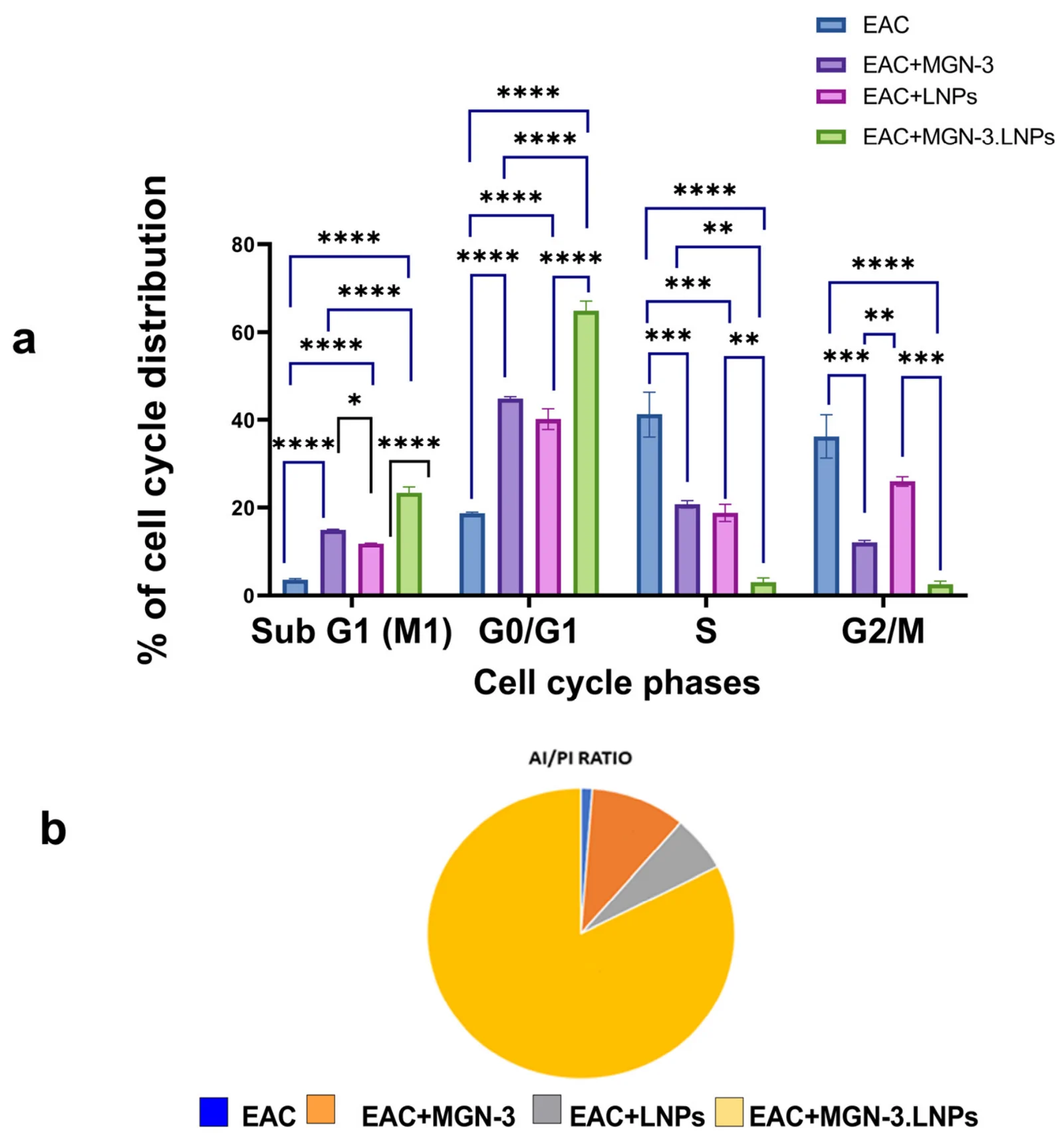
Effects of free MGN-3, plain-LNPs, and MGN-3.LNPs treatments on EAC tumor cell cycle dynamics and cell death kinetics. (**a**) Percentage distributions of tumor cells across distinct cell cycle phases (Sub-G1, G0/G1, S, and G2/M). (**b**) Apoptotic Index to Proliferative Index (AI/PI) ratio expressed as a percentage change relative to the untreated EAC control group. Data are presented as mean ± SEM (*n* = 6 mice/group, representing a randomized sub-cohort selected for flow cytometric analysis). (*, **, ***, ****) indicate statistical significance between groups at *p* < 0.05, *p* < 0.01, *p* < 0.001 and *p* < 0.0001 levels, respectively.

Cell cycle progression was measured via flow cytometry of PI-stained cells. The data in Figure 7a show a highly significant increase (*p* < 0.0001) in hypodiploid cell percentages in the sub-G1 phase, signifying apoptosis for free MGN-3, plain LNPs, and MGN-3.LNPs groups by 313.76% (4.1 fold), 227.06% (3.3 fold) and 544.5% (6.5 fold), respectively, relative to control. Different treatments also arrested the cell cycle in the G0/G1 phase, with a significantly (*p* < 0.0001) increased cell population in the G1 phase (139.75%, 114.97% and 246.43%, respectively) relative to control, followed by a reduction in values for the S and G2/M phases relative to control. A change in Apoptosis Index relative to the Proliferation Index (AI/PI ratio) was also noted. In comparison with the control, groups treated with free MGN-3, plain LNPs and MGN-3.LNPs showed AI/PI ratios increased by (748.44%, 422.46%) and (6891.72, *p* < 0.0001), respectively, as indicated in Figure 7b.

### 2.6. Quantitative Analysis of Apoptosis by Annexin-V/PI Staining

**Figure 8:**
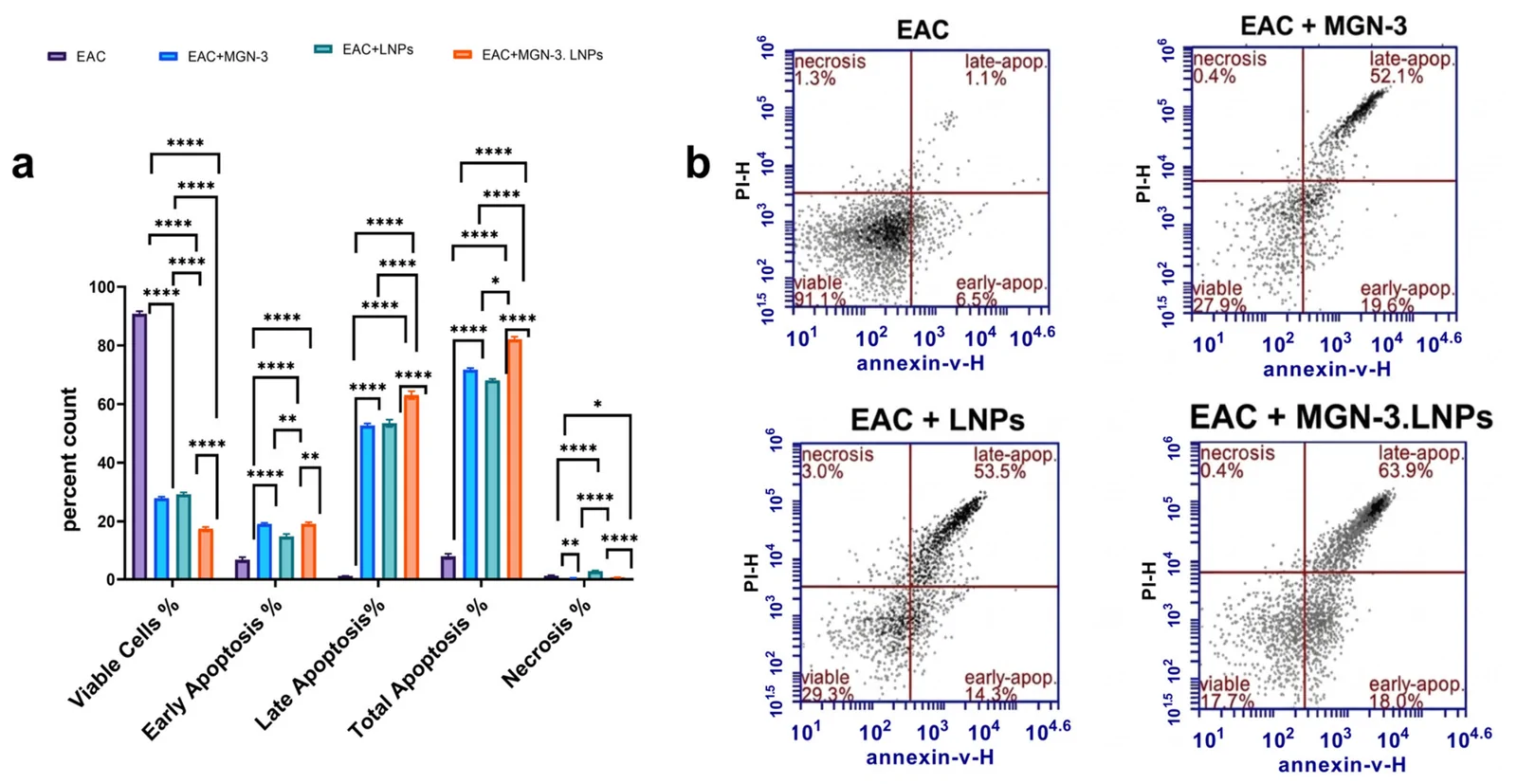
Effect of MGN-3, plain LNPs and MGN-3.LNPs treatment as detected by Annexin-V/PI. (**a**) The percentage count of Annexin V phases. Each value represents the mean ± SEM (5 mice/group). (*, **, ****) indicate statistical significance between groups at *p* < 0.05, *p* < 0.01 and *p* < 0.0001 levels, respectively. (**b**) Representative Annexin-V/PI labeling of tumor cells of MGN-3, plain lipid nanoparticles and MGN-3.LNPs groups as analyzed by flow cytometry, and different experimental groups are labeled with Annexin-V/PI. The lower right (**LR**) quadrant of the histogram displays early apoptotic cells stained with Annexin V-conjugated Alexa flour 488 dye, while the upper right (**UR**) quadrant displays late apoptotic cells. The histogram’s lower left (**LL**) quadrant displays viable cells, whereas the upper left (**UL**) quadrant displays necrotic dead cells.

Apoptosis was quantitatively evaluated via flow cytometry following Annexin V/PI double staining (Figure 8a,b). Relative to the control group, treatment with free MGN-3, plain LNPs, and MGN-3.LNPs significantly reduced cell viability by 69.4%, 67.9%, and 81.1%, respectively (*p* < 0.0001). This cytotoxicity was primarily mediated by programmed cell death; early apoptotic populations increased by 184.5%, 118.2%, and 184.8% relative to control levels, while late apoptotic populations showed massive elevations of 4517.5%, 4587.7%, and 5431.6% (*p* < 0.0001). Consequently, the overall total apoptotic populations expanded by 9.2-fold (free MGN-3), 8.7-fold (plain LNPs), and 10.5-fold (MGN-3.LNPs; *p* < 0.0001) compared to control. Interestingly, necrotic cell populations exhibited divergent trends: plain LNPs significantly increased necrosis by 120.6% (*p* < 0.0001), whereas free MGN-3 and MGN-3.LNPs significantly reduced necrotic cell populations by 63.5% (*p* < 0.001) and 46.03% (*p* < 0.05), respectively.

### 2.7. Cell Cycle Progression and the Expression of Apoptotic Regulatory Proteins

Flow cytometric analysis (Table 3) revealed that protein expression levels for P53, P27, Bax, Caspase-3 and Caspase-9 were markedly upregulated, while the expressions of Bcl2 and ROS were markedly downregulated after treatments. Also, the different treatments led to a large increase in the Bax/Bcl2 ratio relative to control. The expressions of different molecular markers in tumor cells were more marked in the MGN-3.LNPs group, followed by MGN-3 and plain LNPs groups, versus the untreated control group.

**Figure 9:**
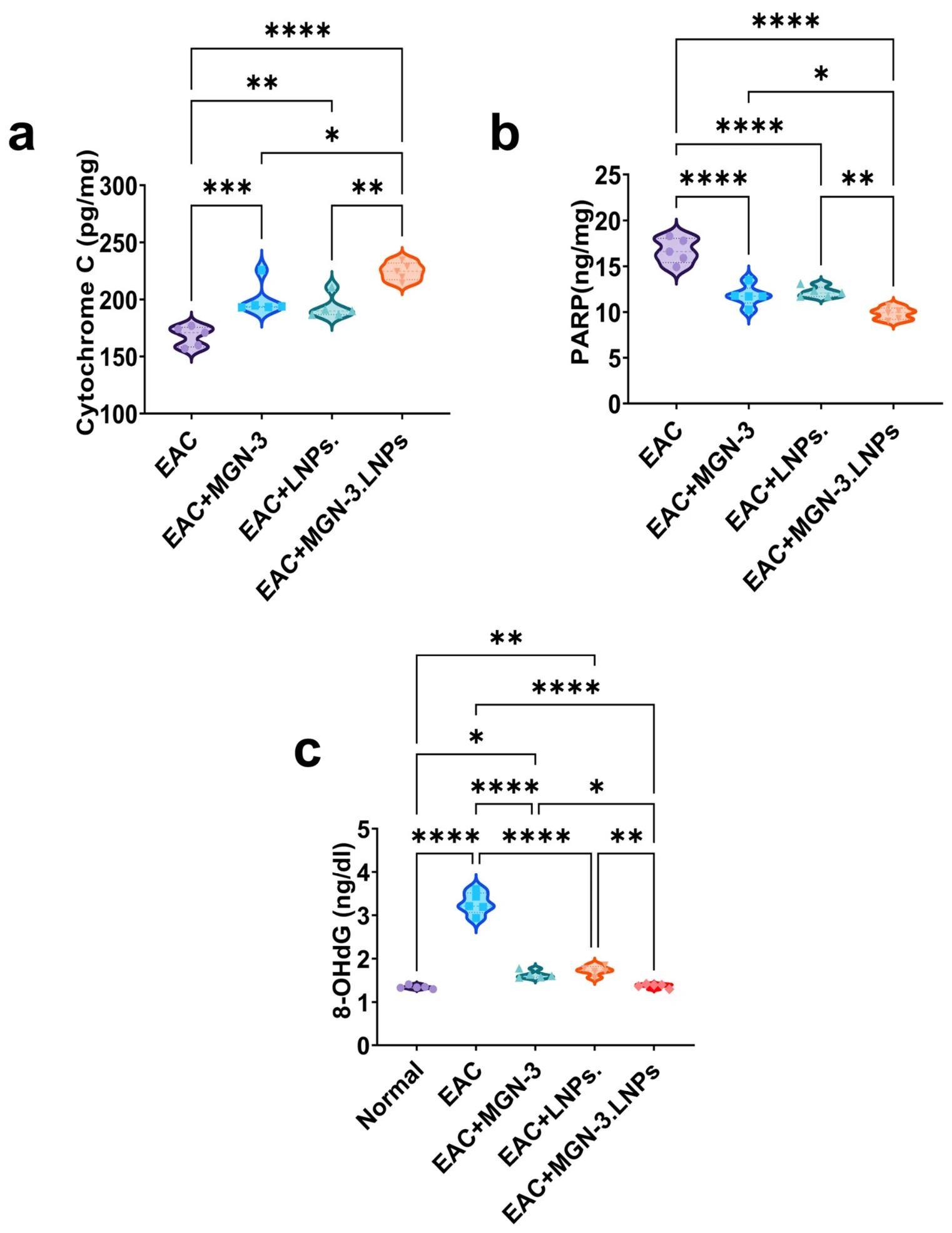
Impact of free MGN-3, plain LNPs, and MGN-3.LNPs on apoptotic signaling markers and systemic oxidative DNA damage. (**a**) Cytochrome c (Cyt c) concentration in solid tumor tissue homogenates. (**b**) Poly(ADP-ribose) polymerase (PARP) expression levels in solid tumor tissue. (**c**) Serum 8-hydroxy-2′-deoxyguanosine (8-OHdG) levels**.** Each value represents the mean ± SEM of 5 mice/group. (*, **, ***, ****) indicate statistical significance between groups at *p* < 0.05, *p* < 0.01, *p* < 0.001 and *p* < 0.0001 levels, respectively.

In addition, the estimation of PARP and cytochrome c levels by ILISA technique showed that the administration of free MGN-3, plain LNPs and MGN-3.LNPs to tumor-bearing mice led to a marked, significant elevation in cytochrome c level and a marked, significant decrease in the level of PARP relative to control. However, the effect was more efficient for MGN-3.LNPs treatment relative to the other treatments (Figure 9a,b). These data indicate that lipid nanoparticles encapsulating MGN-3 were superior to the free MGN-3 and plain LNPs in regulating the cell cycle and apoptotic regulators in tumor cells, indicating its effectiveness in suppressing tumor growth.

### 2.8. Detection of Serum 8-OHdG Level

The level of 8-OHdG as an oxidative DNA damage biomarker was estimated in serum using the ELISA technique. As illustrated in Figure 9c, different treatments markedly decreased the serum levels of 8-OHdG, (*p* < 0.0001) relative to the untreated EAC group, reducing them to within the normal range. However, treatment with MGN-3-.LNPs led to a more marked decline (57.95%), followed by free MGN-3 (50.35%) then plain LNPs (47.27%).

### 2.9. Quantitative Determination of TNF-α and IL-6 Gene Expression by RT-PCR

**Figure 10:**
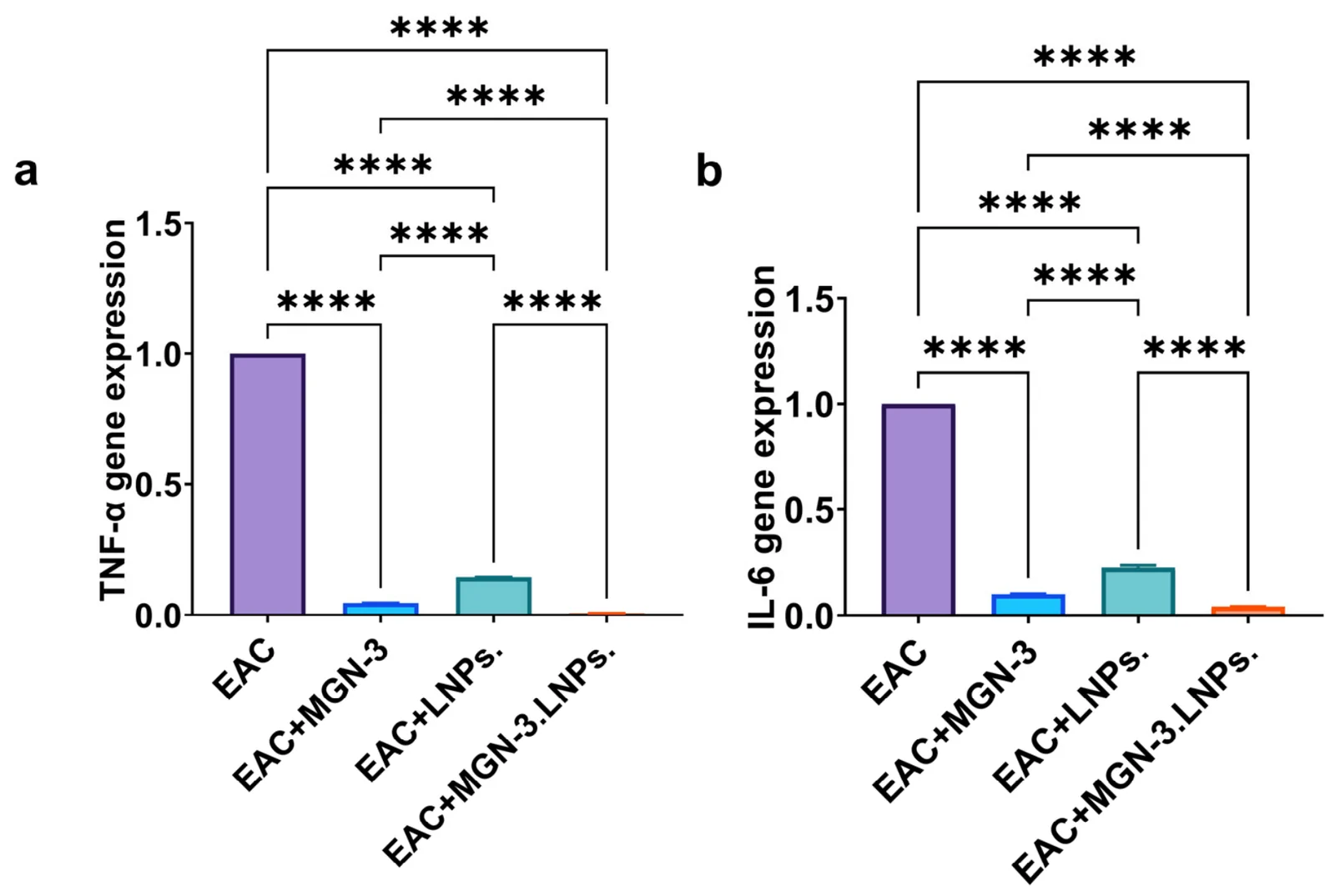
Quantitative real-time PCR (qRT-PCR) analysis of inflammatory cytokine expression. Relative mRNA levels of (**a**) tumor necrosis factor-alpha (TNF-α) and (**b**) interleukin-6 (IL-6) in EAC tumor tissue across experimental groups. Target gene expression was normalized relative to the endogenous housekeeping gene (GAPDH). Data are presented as mean ± SEM (*n* = 5 mice/group). **** *p* <0.0001 indicates high statistical significance compared to the untreated control group.

Real-time quantitative PCR (RT-qPCR) was utilized to evaluate the intratumoral gene expression of the inflammatory markers TNF-α and IL-6 (Figure 10a,b). All treatment regimens induced significant downregulations of both TNF-α and IL-6 relative to untreated EAC control mice (*p* < 0.0001). Notably, MGN-3.LNPs demonstrated superior suppressive efficacy, achieving a significantly greater inhibition of both cytokines compared to free MGN-3 and plain LNPs.

### 2.10. Detection of Oxidative Stress Antioxidants in Tumor Tissues

**Figure 11:**
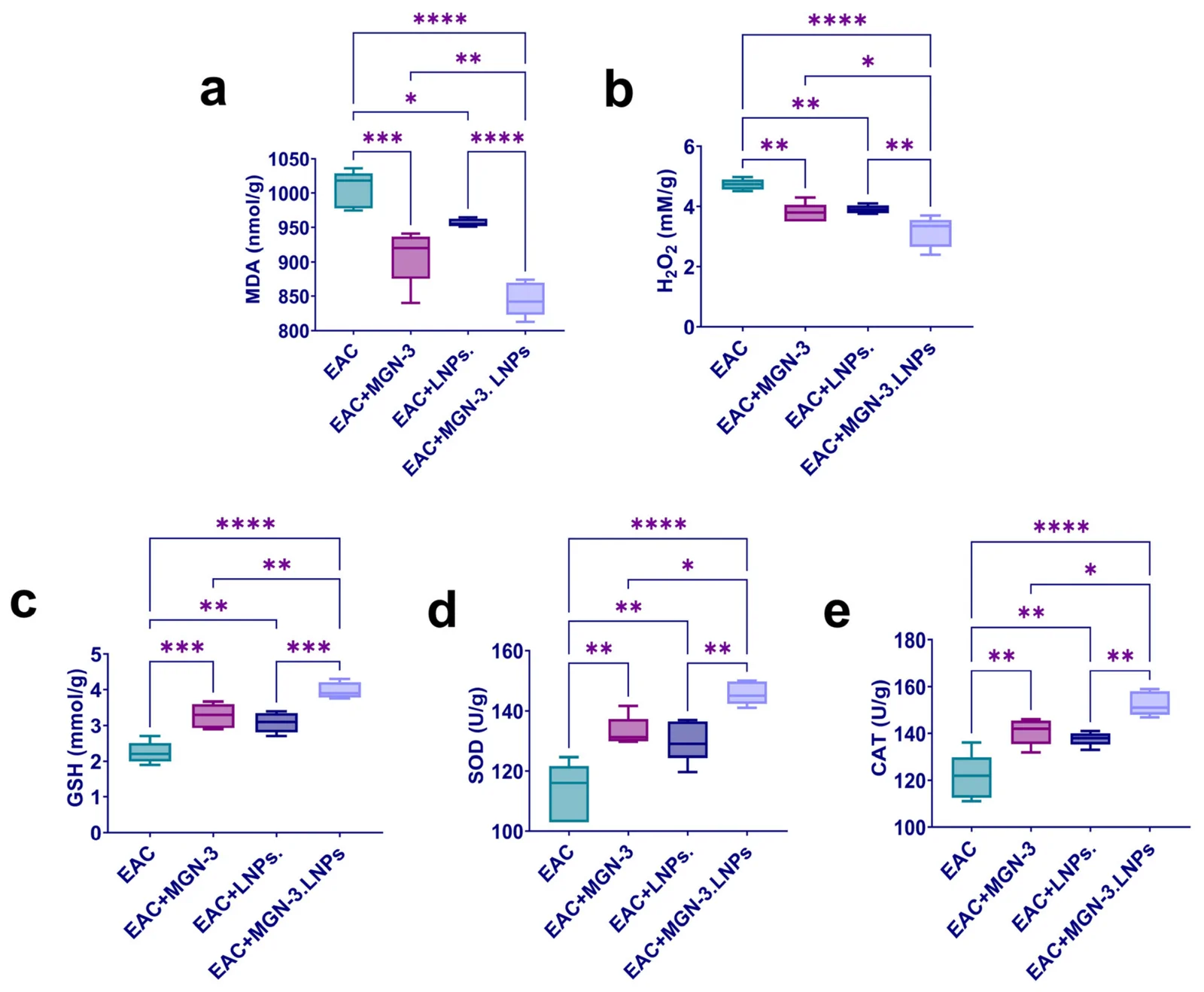
Impact of MGN-3.LNPs on oxidative stress biomarkers and antioxidant capacity in tumor tissues. Levels of (**a**) malondialdehyde (MDA, nmol/g), (**b**) hydrogen peroxide ($H_{2}O_{2}$, nmol/g), (**c**) reduced glutathione (GSH, mmol/g), (**d**) superoxide dismutase activity (SOD, U/g), and (**e**) catalase activity (CAT, U/g) across experimental groups. Data are expressed as Mean± SEM (5 mice/group). (*, **, ***, ****) indicate statistical significance between groups at the *p* < 0.05, *p* < 0.01, *p* < 0.001 and *p* < 0.0001 levels, respectively.

The effects of free MGN-3, plain LNPs, and MGN-3.LNPs on intratumoral oxidative stress and antioxidant biomarkers are presented in Figure 11a–e. Tumor-bearing mice treated with free MGN-3, plain LNPs, or MGN-3.LNPs exhibited significant reductions in pro-oxidant MDA levels (by 9.69%, 4.9%, and 16%, respectively) and $H_{2}O_{2}$ levels (by 19.9%, 17.3%, and 33.3%, respectively) relative to untreated EAC controls. Conversely, antioxidant defenses were markedly elevated following treatment with free MGN-3, plain LNPs, and MGN-3.LNPs, showing increased levels of GSH (by 46.1%, 37.6%, and 77.4%), CAT (by 16%, 13.5%, and 25.8%), and SOD (by 17.7%, 15.1%, and 29%), respectively. Collectively, these findings highlight the superior efficacy of MGN-3.LNPs in mitigating oxidative stress and augmenting antioxidant defenses in tumor tissues compared to free MGN-3 or plain LNPs.

## 3. Discussion

The current study aimed to enhance the anticancer efficacy of MGN-3/Biobran against breast cancer tumor growth and proliferation in vivo via use of a lipid-based nanoencapsulation made from a blend of cinnamon and avocado oils. Our target was to augment the drug bioavailability to reach the target cancer cells more effectively and to elucidate the underlying mechanism. Cinnamon oil’s principal compound (cinnamaldehyde) exerts anticancer activities by altering tumor cell growth, tumor cell proliferation, and tumor cell apoptosis and necrosis [15,16]. In addition, cinnamon oil was shown to act as an efficient nanocarrier [33]. Avocado oil, on the other hand, is a versatile, nutrient-dense oil extracted from the pulp of the avocado fruit. Avocado oil is increasingly used as a natural, edible oil in nanocarrier systems, particularly as an oil phase in nanoemulsions and nanocapsules, due to its effect of increasing drug bioavailability and its inherent antioxidant properties [34]. Recently, MGN-3/Biobran encapsulated in nanofiber was shown to be an effective bioactive material that could augment wound-healing [35,36]. The MGN-3/Biobran was released from the nanofiber scaffolds in a sustained, controlled manner, with Biobran’s release rate decreasing as its concentration increased. The authors concluded that MGN-3/Biobran-loaded core/shell nanofiber scaffolds could be utilized as ideal multipurpose wound dressings for healing wounds. 

Intraperitoneal (IP) chemotherapy for breast cancer demands nanocarriers optimized for maximal peritoneal retention and lymphatic uptake while minimizing systemic off-target clearance [37,38]. Lipid nanoparticles (LNPs) fulfill these requirements by providing superior tumor targeting, site-specific payload release, and high biocompatibility [37,38]. Synthesis via the solvent-free, low-energy phase inversion temperature (PIT) method comprising three heating–cooling cycles (~85 °C to ~55 °C) followed by a 4 °C water shock yields a rigid non-ionic surfactant shell enclosing a lipophilic core [39,40]. This architecture affords high lipophilic drug loading, extended peritoneal residence [38,41], and reduced local inflammation and hypersensitivity [42]. Biocompatible lipid nanocapsules (LNCs) and nanoemulsions (NEs) (20–100 nm) prepared via PIT represent a “smart generation” of nanocarriers with broad applications in biological barrier penetration, targeted delivery, theranostics, and gene therapy [43]. While the use of PIT for solid lipid nanocapsules (SLNCs) with volatile oils remains limited [44], incorporating Tween 20 yields smaller particle sizes and lower polydispersity indices (PDI) than Tween 60 or 80 [44]. Furthermore, liquid paraffin-core NEs demonstrate superior thermal stability over SLNCs (60 °C vs. 50 °C) [45]. The liquid core maximizes lipophilic loading capacity, while the surfactant shell remains solid during storage and melts at body temperature (~37 °C) to provide controlled drug release kinetics [45].

Conventional nanocarriers exhibit significant drawbacks in IP delivery [46,47]. Solid Lipid Nanoparticles (SLNs) suffer from core crystallization during storage, triggering premature drug expulsion and erratic toxicity [46]. Liposomes display low physical stability, rapid enzymatic leakage, and restricted penetration into dense tumor nodules or narrow lymphatics due to their rigid, larger structure [47]. Conversely, polymeric nanoparticles provide sustained release over weeks, but require toxic organic solvents [48] and produce acidic degradation byproducts that cause local tissue irritation and cellular aggregation [49]. Lipid-based nanocarriers (SLNs, LNCs, NLCs) overcome these limitations by promoting tissue permeation and systemic availability while minimizing local tissue accumulation [48]. Incorporating Tween 20, which offers lower toxicity and enhanced lipid compatibility compared to Tween 80 [50], imparts deformability to NEs/LNCs, enabling them to squeeze through 50–100 nm endothelial fenestrations to enhance tumor extravasation [51] and deep spheroid penetration [52]. Overall, solvent-free PIT-LNCs pair a liquid core with a rigid surfactant lecithin shell, delivering high lipophilic payload capacity and polymeric-level stability without risks of acidic degradation or storage-induced aggregation. Physicochemical characterization revealed that MGN-3 core incorporation initially exerted a distinct compacting effect, decreasing the mean hydrodynamic diameter from 106.1±52.2 nm (plain LNPs) to 96.6±33.7 nm (MGN-3.LNPs) and narrowing size distribution linearity ($R^{2}$ increased from 0.64 to 0.93). This matrix contraction confirms successful internal payload integration driven by hydrophobic packing, hydrogen bonding, and electrostatic cross-linking. Transmission electron microscopy (TEM) corroborated dynamic light scattering (DLS) measurements, displaying discrete, uniform spherical morphologies; a geometry highly favorable for cellular endocytosis [53,54]. Despite maintaining a sub-100 nm size profile optimal for passive tumor targeting via the enhanced permeability and retention (EPR) effect and lymphatic uptake, the formulation exhibited a net negative surface charge [54]. This persistent negative charge facilitates cell membrane interactions and intracellular accumulation in solid tumors [54], contrasting with outcomes reported for several other lipid platforms [43,46,55].

Long-term colloidal stability over 18 months at 4 °C (Figure 1) was achieved through a novel dual layer steric barrier; the polyoxyethylene corona of Tween 20 acts synergistically with the hydrophilic arabinose and xylose side chains of MGN-3 (a highly branched arabinoxylan polysaccharide) [52,53]. Extending into the continuous aqueous phase, this dense hydrated shell generates short-range hydration and steric repulsive forces that overcome van der Waals attraction, preventing hydrophobic aggregation and coalescence despite relatively low net negative zeta potentials (−5.58 to −8.22 mV) [53,56].

In vitro dissolution profiles demonstrated biphasic release kinetics for MGN-3.LNPs, contrasting sharply with the rapid burst dissolution of free MGN-3 (99% within 8 h). The nanoformulation liberated ~50% of its active payload within 24 h, followed by sustained release reaching ~98% over 24 days. This profile provides an immediate therapeutic dose from the un-entrapped fraction, accelerating treatment onset, while the core-encapsulated drug reservoir ensures long-term therapeutic efficacy [57]. These findings correlate with observations by El-Far et al. (2023) [58], wherein the nanoencapsulation of Quercetin significantly prolonged drug release compared to the free drug, confirming that nanoformulation enhances bioavailability and formulation stability. Freshly prepared formulations were used prior to in vivo administration to preserve these structural and release characteristics [53].

Upon IP administration, which tempers immediate, high-density plasma exposure compared to direct intravenous boluses [59], the non-ionic Tween 20 shell provides steric stabilization that suppresses non-specific protein opsonization and reticuloendothelial system (RES) clearance [60,61]. Concurrently, the hydrophobic core enriched with natural fatty acids from cinnamon and avocado oils selectively recruits endogenous dysopsonins (e.g., serum albumin and apolipoproteins). Rather than hindering uptake, this dynamic biomolecular protein corona creates a stealth-like biological identity that promotes receptor-mediated endocytosis into tumor cells via lipid/lipoprotein pathways, thereby maintaining systemic nanoparticle integrity and maximizing the intracellular bioavailability of the encapsulated MGN-3 payload [61].

Our data demonstrate that MGN-3.LNPs dramatically decrease tumor growth (TV and TW) relative to EAC-bearing tumors groups that received either free MGN-3 or plain LNPs. A profound retardation in TV was recorded in the group treated with MGN-3.LNPs of controls, relative to free MGN-3 and plain LNPs groups, as early as day 15 after tumor cell inoculation, and TV&TW showed a drastic reduction in controls on day 26. Our earlier findings show that free MGN-3 caused a remarkable delay in both TV&TW in EAC-bearing mice [22]. However, in the present data, the MGN-3-loaded lipidic nanocarrier shows superior antitumor efficacy relative to free MGN-3. Our results are in accord with those of Othman et al. (2017) [62], who observed that natural product-loaded chitosan nanoparticles remarkably decreased tumor growth (TW and TV) in EAC-bearing mice that received free drug and void nanocapsules. Similar trends were also demonstrated elsewhere [58,63]. This potent antitumor activity of MGN-3.LNPs in our study could be attributed to the test compound’s sustained release from LNP capsules, granting a more effective delivery of acceptable concentrations of the entrapped drug to tumor tissues after administration. This treatment mode of MGN-3.LNPs could also allow for efficient antitumor actions that do not simultaneously elicit any notable systemic toxicities [63]. 

Histopathologically, the treatment of EAC-mice with MGN-3.LNPs versus the other treatments yielded the most substantial degeneration in neoplastic cell proliferation, more marked leucocytes infiltration, abundant apoptotic neoplastic cell residues with apparent pyknotic or karyorrhexis nuclei and marked fatty degeneration. These findings confirm the superior antitumor efficacy of MGN-3.LNPs when compared to either free MGN-3 or plain LNPs. 

A hallmark of cancer cells is the dysregulation of the cell cycle, enhancing cellular proliferation. The mechanisms by which MGN-3.LNPs induce regression in tumors in EAC-bearing mice may involve the arrest of the cell cycle and reductions in the qualities of the nanosized agent that enhance apoptosis in cancer cells. Earlier studies reveal that free MGN-3 was found to inhibit hepatocarcinogenesis in rats by inducing apoptosis, and arrested liver cancer cells in the cell cycle’s sub-G1 phase [26]. In this study, an analysis of the progression of the cell cycle’s progression showed the viable cell count to yield the greatest inhibition. Moreover, data on the distribution of the cell cycle show that MGN-3.LNPs treatment led to a marked increase in cell numbering in the G0/G1 phase, together with a decrease in other phases’ cell numbers. In addition, the highest elevation in the sub G0/G1 phase indicated apoptosis induction. This result was confirmed by the greatest increase in AI/PrL ratio, and further indicated by the Annexin V/PI assay that showed that the overall total apoptosis was more pronounced in the nanosized MGN-3.LNPs treatment versus plain LNPs and free MGN-3. This primarily resulted from improved MGN-3 stability and sustained release from the LNP carrier. Other studies have also demonstrated apoptosis induction resulting from the application of cinnamon oil in leukemia cells via the mitochondrial pathway [64], as well as apoptosis induction derived from the effect of a Mexican avocado lipid-rich extract in Caco-2 human colon cancer cells [65].

Targeting the cell cycle and apoptotic mechanism is a new strategy for treating cancer. Molecular analysis shows that the percentages of protein levels of p53 and p27 in cancer cells of mice treated with free MGN-3, plain LNPs, and MGN-3.LNPs were upregulated in comparison to those of the untreated EAC control group and MGN-3. The LNPs group was superior to other treatments. In tumor cells, the tumor suppressor p53 acts as a transcription factor, initiating cell cycle arrest and apoptosis, while p27 inhibits cell proliferation by blocking cyclin-dependent kinases (CDKs), acting as a brake on cell cycle progression. Ki-67 is a nuclear protein widely regarded as an excellent marker for assessing cell proliferation. The higher the expression of Ki-67, the greater the proliferation of cells [66], and the possibly high expression of Ki-67 in the neoplasm indicates cell division and proliferation. In the current study, the proliferation marker of the Ki-67 protein was overexpressed in the untreated tumor tissue, and in contrast, the expression was downregulated in tumor tissues of mice that received various treatments, but the effect was accentuated for those treated with MGN-3.LNPs, indicating the advantages of nanosized encapsulation, which also supports the remarkable decrease in tumor growth observed here. Further, we have investigated the abilities of the different treatments to target several key regulatory molecules that control the induction of apoptosis in tumor cells, including Bax, Bcl-2, caspace-9 and caspase-3 versus untreated controls. Treatment with MGN-3.LNPs was more profound compared to the free MGN-3 and plain LNP in upregulating the expression of Bax, in downregulating the expressions of ROS and Bcl-2 with an increase in the ratio of Bax/Bcl-2, in activating caspase-9 and caspase-3 expression in neoplastic cells, and in increasing serum cytochrome c levels and decreasing PARP levels. Such results indicate that the different treatments induced apoptosis via the intrinsic mitochondrial pathway. The upregulation of caspase-9 and caspase-3 and the changes in Bax/Bcl-2 led to mitochondrial membrane destabilization, the liberation of proteolytic caspases, and PARP cleavage [67], leading to cell death via the intrinsic mitochondrial pathway. 

Likewise, earlier studies revealed that MGN3/Biobran-induced apoptosis results from a mitochondrial pathway [29]. The role of Biobran in activating caspases-9, -8, and -3 and in inducing apoptosis has been shown in vitro [68]. Cleaved caspase-3 is a critical executioner protease in apoptosis, indispensable to DNA fragmentation and chromatin condensation [69]. In addition, different treatments markedly decreased the level of serum 8-OHdG, and downregulated reactive oxygen species (ROS) expression in tumor cells relative to the untreated EAC group. This was found to be more prominent under the MGN-3.LNPs treatment. Cancer cells can produce high ROS levels, which cause oxidative DNA damage, resulting in elevated 8-OHdG levels. High ROS rates have been noted in cancer types, leading to tumor growth and progression [70]. Conversely, anticancer agents that also have antioxidant properties can neutralize these ROS, thereby reducing DNA oxidative stress and lowering 8-OHdG expression [71]. 

Free MGN-3-induced oncostatic activity was previously shown to inhibit tumor growth by protecting against oxidative stress, augmenting the antioxidant defense system, and modulating lipid peroxidation. According to [24], MGN-3 suppressed tumor growth by normalizing MDA and H_2_O_2_ levels, augmenting GSH contents and enhancing the activity of the endogenous antioxidant-scavenging enzymes SOD, GPx, and CAT in tumor tissues of mice bearing tumors. The current study yielded the same results in tumor tissues; however, the nanosized MGN-3-loaded LNPs were apparently more potent in modulating oxidative stress and augmenting antioxidants versus free MGN-3. This primarily resulted from improved MGN-3 stability and the sustained release from LNP capsules, which may have allowed for the sufficient release of entrapped drug concentrations to tumor tissues after administration. It is interesting that plain LNPs in the current study revealed a notable anticancer activity. The main active ingredients of cinnamon oil (cinnamic acid, eugenol, and cinnamaldehyde) are recognized for their anti-tumorgenesis role that induces apoptosis in tumor cells and has antioxidant properties [72,73,74,75]. Also, avocados contain many anticancer nutrients, and avocado phytochemicals have the potential to inhibit cell proliferation and stimulate apoptosis [76].

Emerging research highlights the critical roles of oxidative stress and inflammation in cancer development, as inflammatory responses serve as key mechanisms driving cancer promotion and tumorigenesis [77]. Treatment with free MGN-3, plain LNPs and MGN-3.LNPs yielded a considerable down regulation of TNF-α and IL-6 relative gene expression versus untreated EAC mice. However, MGN-3.LNPs’ anti-inflammatory effectiveness was superior to those of free MGN-3 and plain LNPs in inhibiting the expressions of both TNF-α and IL-6 genes. MGN-3/Biobran was shown to act as a potent immunomodulator that exhibits significant anti-inflammatory effects. MGN-3 inhibits liver cancer development triggered by carcinogens like NDEA plus CCl4 by promoting cancer cell death while reducing inflammation and stopping tumor growth [26]. In addition, scientific studies indicate that cinnamon oil and its active components exhibit both anti-inflammatory and potent anti-cancer effects in various cancer cell lines and animal models [78].

## 4. Materials and Methods

### 4.1. Chemicals

MGN-3 (Biobran; Daiwa Pharmaceutical Co., Ltd., Tokyo, Japan) is a low-molecular-weight (30–50 kDa) denatured arabinoxylan produced by reacting rice bran hemicellulose with carbohydrate-hydrolyzing enzymes derived from Shiitake mushroom (*Lentinula edodes*) mycelia [19]. Structurally characterized by a β-(1,4)-D-xylopyranose backbone decorated with α-L-arabinofuranose side chains, MGN-3 features an arabinose-to-xylose (A/X) ratio of approximately 0.5 (~1:2), providing a moderately branched configuration that imparts optimal water solubility and enhanced biological accessibility compared to intact native polysaccharides [25]. Crucially, despite its overall hydrophilic character, localized hydrophobic domains along the non-polar axial faces of the pyranose rings and ester-linked ferulate residues enable robust non-covalent hydrophobic interactions with the essential oil lipid core (cinnamon and avocado oils) [79], ensuring efficient loading and stable encapsulation within the lipid nanoparticle matrix.

Avocado oil: Oleic and linoleic acids are the main fatty acids found in avocado oil. Avocado oil was defined by a high lipid content of 62%, with a higher proportion of oleic (42–51%) and palmitic (20–25%) lipids. OOO (21–34%) and OOP (19–24%) were two of the most common triacylglycerols; O and P stand for oleic and palmitic acids, respectively [80]. Avocado oil was provided by Albadawia company, Mansoura, Egypt.

Cinnamon oil: The main chemicals identified in cinnamon oil are 1,8-cineole; terpinen-4-ol; camphor; linalool; α-cadiene; α-terpineol; germacrene D; α-cadinol; γ-muurolene; safrole; eugenol; 1-phenyl-propanr-2,2-diol diethanoate; and 1,6-octadien-3-ol,3,7-dimethyl [81]. Cinnamon oil was provided by Albadawia company, Mansoura, Egypt.

Span 60: Sorbitan monostearate (Span 60) is synthetic wax that is used as an emulsifier to maintain the mixing of water and oils. Span 60 is a non-ionic, entirely bio-based surfactant that is utilized as a W/O emulsifier [82]. Span 60 was acquired from Sigma-Aldrich.

### 4.2. Preparation of Lipidic Nanoparticles by Inversion Method

Oil-in-water nanoemulsions can be created using low-energy techniques such as phase inversion temperature (PIT). This approach includes variations in surfactant solubility or phase transitions in their physicochemical characteristics due to temperature changes [83,84]. The phase inversion was used to formulate all lipid nanocapsule (LNC) formulations. The LNCs were made utilizing span 60 and two distinct oils: avocado and cinnamon. Through constant stirring at 250 rpm and 50 °C, equivalent amounts of oils and surfactant (Avocado:Cinnamon:span; 1:1:1) were combined to create the oil phase. Additionally, 2.5 mL of the aqueous phase was heated to 70 °C before being added dropwise to the oil phase. After cooling the prepared LNCs, 1 mL of cooled distilled water was added; the combination was slowly and continuously stirred for 15 min to perform O/W to W/O phase inversion. The same process was used to prepare drug-loaded LNCs (MGN-3-LNCs); MGN-3.LNCs were prepared using powder MGN-3, span 60 and two distinct oils—avocado and cinnamon. Under constant stirring at 250 rpm and 50 °C, equivalent amounts of oils and surfactant (avocado:cinnamon:span; 1:1:1—0.5 mL from each) were combined to create the oil phase. Additionally, 50 and 70 mg of MGN-3 were dissolved in 2.5 mL of distilled water then heated to 70 °C before being added dropwise to the oil phase. After cooling the prepared MGN-3.LNCs, 1 mL of cooled distilled water was added, and the combination was slowly and continuously stirred for 15 min to perform O/W to W/O phase inversion, while the size and entrapment efficiency percentage measurements were used to determine the ideal MGN-3 dosage. When compared to 100% solid lipid, the MGN-3 final total concentration was 10% *w*/*w*. The resultant solution was stored in the refrigerator. The size and entrapment efficiency percentage measurements were used to determine the ideal drug dosage.

### 4.3. Characterization of Plain and MGN-3-Loaded Lipidic Nanoparticles

#### 4.3.1. Measurement of the Particle Size, Polydispersity Index (PDI), and Formulation Surface Charge

The size and zeta potential of plain and MGN-3-loaded LNCs were measured with Zetasizer (Malvern Instruments Ltd., Worcestershire, UK) at various time intervals following storage in order to assess the stability of LNCs. The size and shape of MGN-3-loaded LNCs were measured with transmission electron microscopy (TEM- JEOL, JEM-1230, Tokyo, Japan) at an accelerating voltage of 0 kV. 

#### 4.3.2. Evaluating the Drug Entrapment Efficiency, Loading Capacity and In Vitro Cumulative Drug Release

The drug entrapment efficiency (EE%, Equation (1)) and loading capacity (LC%, Equation (2)) of the prepared MGN-3-loaded LNPs were measured by centrifuging them for 10 min at 40 °C and 30,000 rpm. A UV-Vis spectrophotometer (Evolution UV 600, Thermo Scientific, Waltham, MA, USA) was then used to measure the amount of free MGN-3 in the supernatant at a wavelength of 250 nm. Every calculation and experiment was carried out three times. The following formulas were used to determine the medicines’ entrapment efficiency (EE%) and loading capacity (LC%):

(1) $$EE\%=\left(\frac{Total\;drug-Free\;unloaded\;drug}{Total\;drug}\right)\times 100$$

(2) $$LC\%=\left(\frac{Total\;drug-Free\;unloaded\;drug}{Weight\;of\;drug\;loaded\;Nanoparticles}\right)\times 100$$

The release of MGN-3 from LNPs was carried out over 24 days under tumor physiological conditions—37 °C and PH 7 in dynamic phosphate buffer saline. MGN-3 and MGN-3-LNPs were encapsulated in a dialysis bag (cut-off; 14 kDa) immersed in 2 mL of the release media. At predetermined time intervals, the release media was removed and replaced with a fresh solution. Then the withdrawn aliquot was used to determine the amount of drug released using a UV-Vis spectrophotometer.

All experiments were performed in triplicate to ensure accuracy and reproducibility, and the cumulative drug release (%) at each interval was calculated according to Equation (3).

(3) $$C_{n}=C_{n\;means}+\frac{A}{V}\sum_{s=1}^{n-1}C_{s\;means}$$

where A is the volume of the aliquot, V is the volume of the release medium, Cn is the cumulative concentration of all withdrawn samples, *C*_*n* means_ is the calculated concentration, n − 1 is the sum of the former aliquot, and *C*_*s* means_ is the total concentration of all earlier aliquots.

### 4.4. Experimental Animals and Tumor Model

A total of 55 female Swiss albino mice (2 months old, weighing 22–25 g) were obtained from the National Cancer Institute (Cairo University, Egypt). Animals were housed in polypropylene cages (10 mice/cage) under controlled environmental conditions (24±2 °C, 50±10% relative humidity, and a 12 h light/dark cycle) with access to standard pellet food and water ad libitum. Ehrlich Ascites Carcinoma (EAC) cells, sourced from the National Cancer Institute, were maintained in vivo via weekly intraperitoneal passages ($2.5\times 10^{6}\;\mathrm{cells}/\mathrm{mouse}$) in host female Swiss albino mice. To induce solid tumors, a 0.2 mL suspension containing $2.5\times 10^{6}\;\mathrm{viable}\;\mathrm{EAC}\;\mathrm{cells}$ was injected subcutaneously into the lower back of each mouse. All experimental protocols were approved by the Mansoura University Animal Care and Use Committee (Approval Code: MU-ACUC[Sc.MS.23.07.28]).

### 4.5. Experimental Design

Following the subcutaneous inoculation of EAC cells on day 0, palpable solid tumors developed by day 8, at which point treatment protocols were initiated. Interventions were administered via intraperitoneal (i.p.) injection at a fixed volume (e.g., 0.1 mL) per mouse, three times weekly until study termination on day 26 [22]. Free MGN-3 and its nanoformulation were administered at a standardized dosage equivalent to 40 mg/kg body weight. Mice were randomly allocated into five experimental groups:

- Group 1 (Normal Control, *n* = 10)—Untreated, non-tumor-bearing healthy mice;
- Group 2 (EAC Control, *n* = 10)—Tumor-bearing mice receiving no treatment;
- Group 3 (Free MGN-3, *n* = 10)—Tumor-bearing mice treated with free MGN-3;
- Group 4 (Plain LNPs, *n* = 10)—Tumor-bearing mice treated with vehicle plain LNPs;
- Group 5 (MGN-3.LNPs, *n* = 15)—Tumor-bearing mice treated with MGN-3-loaded LNPs.

### 4.6. Evaluation of the Antitumor Activity

#### 4.6.1. Tumor Volume, Tumor Weight, and Sample Collection

Digital Vernier calipers were used to measure tumor volume (TV) at different time intervals beginning on day 8 after EAC inoculation and continuing to day 26. TV (mm^3^) was calculated using 0.52 AB^2^, where A and B are the minor and major axes, respectively.

At the end of the experiment, animals were fasted for 16 h and anesthetized using diethyl ether. Blood was withdrawn from abdominal aorta using vacuum tubes and left to clot at room temperature. Centrifugation at 3000 rpm was used to separate out the serum, which was then stored until being assayed. Animals were sacrificed by cervical dislocation. Solid tumors were excised and their weight (TW/g) was determined. Tumor tissues were either immediately frozen until investigations could be performed or embedded in paraffin for tumor histopathology and processing with immunostaining. 

#### 4.6.2. Histopathological Study

Solid tumor samples from the different groups were collected and promptly preserved for 24 h in 10% neutral buffer formalin. Specimens were dehydrated by an ascending series of ethyl alcohol followed by xylene treatment. The tissues were then embedded in paraffin wax, cut into 4 to 5 μm-thick sections by microtome, mounted on glass slides, dewaxed in xylene, rehydrated in a series of ethanol and stained with HX and Eosin. Histopathological scoring in between the treatment sets was conducted semiquantitatively by a single, non-blinded observer as follows: (−) is nil, (+) is low level observed, (++) is moderate level observed and (+++) is high level observed for leucocytic infiltration, apoptosis and necrosis.

#### 4.6.3. Immunohistochemical Detection of Ki-67 Expression

Paraffin-embedded tumor tissue sections (4 µm) were mounted on charged slides, deparaffinized, rehydrated, and microwaved in 10 mM citrate buffer (pH 6.0) for antigen retrieval. After quenching endogenous peroxidase with $H_{2}O_{2}$ and blocking non-specific sites, sections were incubated with anti-Ki-67 antibody (1:500; cat. no. ab15580; Abcam, Cambridge, UK) overnight at 4 °C. Detection was performed using biotinylated goat anti-rabbit IgG (1:200; cat. no. ab6720; 2 h), streptavidin-HRP (cat. no. ab64269; 10 min), and 3,3′-diaminobenzidine (DAB; 10 min). Sections were counterstained with hematoxylin (1 min) and evaluated via light microscopy. The Ki-67 labeling index (ratio of brown-stained nuclei to total nuclei) was quantified across five random fields per specimen using ImageJ software (v1.42q) [85].

#### 4.6.4. Flow Cytometric Analysis

To prepare single cell suspensions for flow cytometric analysis, tumor tissues were mechanically disaggregated through nylon mesh (40–50 mesh count/cm; HD 140 Züricher Beuteltuchfabrik AG, Rüschlikon, Switzerland) using Tris-EDTA buffer (pH 7.5), centrifuged (200–300× *g*, 10 min), and resuspended in sterile PBS (1 × 10^6^ cells/mL). For cell cycle analysis, ethanol-fixed cells (70% ice-cold, 4 °C) were centrifuged (300× *g*, 5 min) and stained with propidium iodide (PI; 25 µg/mL; Sigma-Aldrich, St. Louis, MO, USA) solution containing 0.1 mM EDTA and 10 µg/mL RNase A in the dark for 30 min to assess DNA phase distribution (G0/G1, S, and G2/M) and the apoptosis-to-proliferation index (AI/PrI) ratio. Apoptosis was quantified in live cells (1 × 10^6^ cells/mL) using an Annexin V-FITC/PI apoptosis detection kit (BD Biosciences, San Jose, CA, USA) following a 15 min room temperature incubation in the dark with 5 µL each of Annexin V-FITC and PI. For cell cycle and apoptotic regulatory protein profiling, tumor cells (1 × 10^6^ cells) were incubated with primary antibodies (1 µg/1 × 10^6^ cells) against p53 (cat. no. sc-98), p27 (cat. no. sc-1641), Bax (cat. no. sc-7480), Bcl-2 (cat. no. sc-7382), caspase-3 (cat. no. IC835G), or caspase-9 (cat. no. ab65615) from Santa Cruz Biotechnology, Inc. (Dallas, TX, USA) for 1 h at room temperature, followed by FITC-conjugated secondary antibody incubation for 20 min and washing with 1% BSA in PBS.

Intracellular reactive oxygen species (ROS) were measured by incubating cells with 2′,7′-dichlorodihydrofluorescein diacetate (DCFH-DA; Sigma-Aldrich) at 37 °C for 30 min in the dark, followed by two PBS washes. All flow cytometric acquisitions (20,000 events/sample) were conducted on a BD Accuri C6 flow cytometer (BD Biosciences) and analyzed using BD Accuri C6 software or FlowJo software (v10.7.2) [86].

#### 4.6.5. Determination of PARP, Cytochrome C and 8-OHdG Levels by ELISA

Intratumoral concentrations of poly(ADP-ribose) polymerase (PARP) and cytochrome c, alongside serum levels of 8-hydroxy-2-deoxyguanosine (8-OHdG), were quantified via enzyme-linked immunosorbent assay (ELISA) according to the manufacturers’ protocols. Excised tumor tissues were weighed, minced, and homogenized in phosphate-buffered saline (PBS, pH 7.4). PARP levels in tissue homogenates were measured using a mouse PARP ELISA kit (cat. no. MBS3806832; MyBioSource, Inc., San Diego, CA, USA). Intratumoral cytochrome c and serum 8-OHdG levels were quantified using specific mouse ELISA kits (cat. nos. CSB-EL006328RA and CSB-E10526r, respectively; Cusabio Technology LLC, Cairo, Egypt). Briefly, standards and samples were added to microplates pre-coated with target-specific capture antibodies, followed by incubation with biotinylated detection antibodies and streptavidin-HRP conjugates at 37 °C for 1–2 h. Following thorough wash cycles, a chromogen substrate was added, and the reaction was quenched with an acidic stop solution. Absorbance was measured at 450 nm using a microplate reader, and target concentrations were calculated by comparing sample optical densities (O.D.) against standard curves run simultaneously.

#### 4.6.6. Quantitative Determination of TNF-α and IL-6 Relative Gene Expression in Tumor Tissue by RT-PCR

TNF-α and IL-6 relative gene expressions in tumor tissue were determined by the RT-PCR method. Total RNA was extracted from tumor cells of the different groups by using an RNeasy Plus Mini Kit (Cat. No. 74104, QIAGEN GmbH, 40724 Hilden, Germany) according to the manufacturer’s instructions. The quality and concentration of RNA were measured by use of a NanoDrop ND-2000 spectrophotometer (Thermo Fisher Scientific, Wilmington, DE, USA). Total RNA was reverse-transcribed (RT) into cDNA using a Thermo Scientific Maxima First Strand cDNA Synthesis Kit (Cat. No. K1641). It contains gDNase, which can remove genomic DNA by incubation at 42 °C for three minutes, protecting the total RNA from genomic DNA interference. The method is in line with the manufacturer’s guidelines. Real-time RT-PCR was conducted using a Thermo Scientific Maxima SYBR Green qPCR Master Mix (Cat. No. K0223) Kit. The reaction conditions and data analysis were conducted in compliance with the manufacturer’s instructions: 2 μL of cDNA in a total volume of 25 μL containing 12.5 μL Maxima SYBR Green qPCR Master Mix (2×), Forward Primer 0.3 μL, and Reverse Primer 0.3 μL. For real-time quantitative PCR, the primers used for respective gene amplifications are shown in Table 4.

The following cycling conditions were used to run each reaction in triplicate on the Thermo Fisher Scientific (PikoReal Real Time Thermal Cycler) (Ratastie, Vantaa, Finland) SN (OR0241300230). Thermal cycling was carried out over 10 min at 95 °C, followed by 40 cycles of [15 s at 95 °C, 30 s at 60 °C, 30 s at 72 °C]. Differences in gene expressions between groups were calculated according to the method in [87] using the $2^{-\Delta \Delta C_{T}}$(cycle time, Ct) method. Differences were normalized against GAPDH and expressed as relative mRNA levels in comparison with controls. 

#### 4.6.7. Determination of Oxidative Stress and Antioxidant Biomarkers in Tumor Tissues

Intratumoral levels of hydrogen peroxide ($H_{2}O_{2}$) and malondialdehyde (MDA) were measured colorimetrically in tumor tissue homogenates as previously described [88,89]. The reduced glutathione (GSH) content was evaluated according to the colorimetric method of [90]. Furthermore, the enzymatic activities of catalase (CAT) and superoxide dismutase (SOD) in tumor homogenates were quantified following the procedures established by [91] and [92], respectively.

### 4.7. Statistical Analysis 

Statistical analyses were performed using GraphPad Prism software (version 9.0; GraphPad Software Inc., San Diego, CA, USA). Data are here expressed as mean ± standard error of the mean (SEM). Intergroup comparisons were evaluated using one-way analysis of variance (ANOVA) followed by the Newman–Keuls post-hoc test. Percentage changes relative to the control group were calculated for each treatment. Statistical significance was established at *p* < 0.05, with higher levels of significance explicitly noted.

## 5. Conclusions

This study demonstrates that encapsulated delivery via lipid-based nanocarriers significantly enhances the anticancer efficacy of the natural product MGN-3/Biobran against breast carcinoma. Nanoencapsulation optimized the biodistribution and cellular uptake of MGN-3, providing the sustained drug release kinetics necessary to maintain therapeutic concentrations over time. Consequently, MGN-3.LNPs exhibited superior antitumor activity against solid Ehrlich Ascites Carcinoma (EAC) growth compared to free MGN-3. Mechanistically, MGN-3.LNPs significantly outperformed free MGN-3 by suppressing tumor cell proliferation, inducing mitochondria-dependent apoptosis, modulating key regulatory protein expression, attenuating oxidative stress, restoring endogenous antioxidant defenses, and downregulating pro-inflammatory cytokine expression. Overall, this lipid nanoparticle platform presents a promising therapeutic strategy to maximize anticancer efficacy while potentially minimizing off-target systemic toxicity.

## Figures and Tables

**Table 1:** Zeta size and potential of plain nanoparticles and MGN-3-lipid nanoparticles loaded with different amounts of MGN-3.

Drug	Amount of MGN-3 (mg)	Size (nm)	PDI	ζ Potential (mV)
Plain nanoparticles	0	234.8 ± 9.38	0.13 ± 0.02	−3.56 ± 1.69
MGN-3-Lipid nanoparticles	50	189.7 ± 5.273	0.106 ± 0.042	−8.22 ± 0.127
MGN-3-Lipid nanoparticles	70	323.5 ± 1.793	0.135 ± 0.038	−2.85 ± 0.205

**Table 2:** The histopathological scoring in between the treatment sets.

	EAC	EAC+MGN-3	EAC+LNPs	EAC+MGN-3.LNPs
Leucocytic infiltration	+	++	++	+++
Apoptosis	+	+++	++	+++
Necrosis	+++	++	+++	++

**Table 3:** Effects of MGN-3, plain LNPs, and MGN-3.LNPs treatments on the expression levels of cell cycle and apoptotic regulator proteins, assessed by flowcytometry.

Parameters	EAC	EAC+MGN-3	EAC+LNPs	EAC+MGN-3.LNPs
P53 expression	23.2 ± 0.82	65.67 ± 1.18 ^B^	40.23 ± 0.48 ^B C^	82.42 ± 0.8 ^B C D^
% changes from EAC-group	-	183.05%	73.42%	255.24%
P27 expression	10.73 ± 0.73	58.42 ± 0.76 ^B^	42.92 ± 1.59 ^B C^	81.42 ± 2.1 ^B C D^
% changes from EAC-group	-	444.25%	299.84%	658.54%
Bax expression	8.85 ± 0.96	62.03 ± 0.89 ^B^	52.83 ± 1.45 ^B C^	84.87 ± 1.25 ^B C D^
% changes from EAC-group	-	600.94%	496.99%	858.95%
Bcl2 expression	80.45 ± 3.52	33.45 ± 1.14 ^B^	59.3 ± 0.65 ^B C^	23.13 ± 0.97 ^B C D^
% changes from EAC-group	-	58.42%	26.29%	71.25%
Bax/Bcl2 ratio	0.1095 ± 0.01	1.863 ± 0.06 ^B^	0.8906 ± 0.02 ^B C^	3.692 ± 0.12 ^B C D^
% changes from EAC-group	-	1601.07%	713.15%	3271.13%
Caspase-3 expression	13.35 ± 0.81	54.65 ± 0.97 ^B^	41.63 ± 1.56 ^B C^	76.37 ± 1.1 ^B C D^
% changes from EAC-group	-	309.36%	211.86%	472.04%
Caspase-9 expression	12 ± 0.57	59 ± 1.29 ^B^	36.32 ± 1.0 ^B C^	74.68 ± 1.23 ^B C D^
% changes from EAC-group	-	391.67%	202.64%	522.36%
ROS expression	70.63 ± 1.04	31.07 ± 1.03 ^B^	37.85 ± 1.18 ^B C^	19.37 ± 0.25 ^B C D^
% changes from EAC-group	-	56.02%	46.41%	72.58%

Data are expressed as Mean ± S.E. (6 mice/group). ^B^ Significantly different from EAC control group at *p* < 0.01. ^C^ Significantly different from treated EAC+MGN-3 group at *p* < 0.01. ^D^ Significant compared to the treated EAC+LNPs group at *p* < 0.01.

**Table 4:** Primers used for respective gene amplifications in the real-time quantitative PCR.

Gene	Forward Primer	Reverse Primer
GAPDH	TGCCACTCAGAAGACTGTGG	GGATGCAGGGATGATGTTCT
TNF-α	TCTTCAAGGGACAAGGCTGC	CTTGATGGCAGAGAGGAGGC
IL-6	TCCTACCCCAACTTCCAATGCTC	TTGGATGGTCTTGGTCCTTAGCC

## Data Availability

Data and materials used and/or analyzed during the current study are available from the corresponding author on reasonable request.

## Supplementary Materials

The following supporting information can be downloaded at: https://www.mdpi.com/article/10.3390/ijms27177953/s1.
